# Histone Acetylation Modifications Affect Tissue-Dependent Expression of Poplar Homologs of C_4_ Photosynthetic Enzyme Genes

**DOI:** 10.3389/fpls.2017.00950

**Published:** 2017-06-08

**Authors:** Yuan Li, Xiu-Mei Dong, Feng Jin, Zhuo Shen, Qing Chao, Bai-Chen Wang

**Affiliations:** ^1^Photosynthesis Research Center, Key Laboratory of Photobiology, Institute of Botany, Chinese Academy of SciencesBeijing, China; ^2^State Key Laboratory of Forest Genetics and Tree Breeding, Northeast Forestry UniversityHarbin, China

**Keywords:** poplar, C_4_ genes, homologs, transcriptional regulation, histone acetylation modification

## Abstract

Histone modifications play important roles in regulating the expression of C_4_ photosynthetic genes. Given that all enzymes required for the C_4_ photosynthesis pathway are present in C_3_ plants, it has been hypothesized that this expression regulatory mechanism has been conserved. However, the relationship between histone modification and the expression of homologs of C_4_ photosynthetic enzyme genes has not been well determined in C_3_ plants. In the present study, we cloned nine hybrid poplar (*Populus simonii* × *Populus nigra*) homologs of maize (*Zea mays*) C_4_ photosynthetic enzyme genes, *carbonic anhydrase* (*CA*), *pyruvate orthophosphate dikinase* (*PPDK*), *phosphoenolpyruvate carboxykinase* (*PCK*), and *phosphoenolpyruvate carboxylase* (*PEPC*), and investigated the correlation between the expression levels of these genes and the levels of promoter histone acetylation modifications in four vegetative tissues. We found that poplar homologs of C_4_ homologous genes had tissue-dependent expression patterns that were mostly well-correlated with the level of histone acetylation modification (H3K9ac and H4K5ac) determined by chromatin immunoprecipitation assays. Treatment with the histone deacetylase inhibitor trichostatin A further confirmed the role of histone acetylation in the regulation of the nine target genes. Collectively, these results suggest that both H3K9ac and H4K5ac positively regulate the tissue-dependent expression pattern of the *PsnCAs*, *PsnPPDKs*, *PsnPCKs*, and *PsnPEPCs* genes and that this regulatory mechanism seems to be conserved among the C_3_ and C_4_ species. Our findings provide new insight that will aid efforts to modify the expression pattern of these homologs of C_4_ genes to engineer C_4_ plants from C_3_ plants.

## Introduction

The C_3_ photosynthetic pathway is the predominant method of carbon fixation and is used by over 90% of terrestrial plant species. C_3_ photosynthesis is relatively inefficient, due to the inefficiency of the carbon-fixing enzyme, Rubisco, which catalyzes both carboxylation (carbon fixation) and oxygenation (photorespiration). The evolution of C_4_ photosynthesis about 35–40 million years ago provided a natural solution to remedy the inefficiency of Rubisco, and in angiosperms C_4_ photosynthesis has emerged independently at least 66 times from C_3_ plants (Sage et al., 2012).

Unlike C_3_ plants, C_4_ plants have a Kranz-type anatomy of altered vascular bundles, and two separate cell types, bundle sheath and mesophyll, are required for photosynthesis (Slack and Hatch, 1967; Hattersley, 1984). The light dependent reactions and the first carbon fixation step occur in the mesophyll cells, whereas the second carbon fixation step by Rubisco occurs in the lower oxygen environment of the bundle sheath cells. In addition to altered anatomical structures, the evolution of C_4_ photosynthesis required changes in the expression of essential enzymes such as PEPC and CA that catalyze the CO_2_ concentration reactions, and PPDK, NADP- or NAD-dependent malic enzymes (NADP-ME or NAD-ME, respectively) and PCK that shuttle four-carbon acids between the mesophyll and bundle sheath cells (Hatch et al., 1975).

All of the enzymes required for the C_4_ photosynthesis pathway are present in C_3_ plants, albeit with much lower activities (Ku et al., 1996). In plants, these C_4_ photosynthetic enzymes or their isoforms are important for central metabolism. β-CA activity is found in the cytosol of mesophyll cell of C_4_ plants, where it catalyzes the first reaction in the C_4_ photosynthesis CO_2_-concentrating mechanisms (CCM), the conversion of atmospheric CO_2_ to HCO3^-^ (Gutierrez et al., 1974; Hatch and Burnell, 1990). In C_3_ plants, β-CA is localized in leaf mesophyll chloroplasts of higher C_3_ plants, where CA can make up 1 to 2% of total leaf protein (Okabe et al., 1984; Fett and Coleman, 1994; Peltier et al., 2006). PEPC is the primary carboxylase in the mesophyll cells, whereas a CO_2_ pump is established in C_4_ plants (Westhoff and Gowik, 2004). In C_3_ plants, PEPC plays a critical role in modulating the balance of carbon and nitrogen metabolism, and amino acid synthesis (Shi et al., 2015). PPDK controls amino acid metabolism and starch biosynthesis in seeds, provides PEP to the shikimate pathway for lignin biosynthesis in the mid-vein of leaves, and plays an important role in the transport of amino acids during natural leaf senescence in *Arabidopsis thaliana* (Taylor et al., 2010). Besides an important role in gluconeogenesis (Leegood and Ap Rees, 1978; Eastmond et al., 2000; Rylott et al., 2003; Malone et al., 2007), PCK also involves in pH stability and nitrogen and amino acid metabolism in many C_3_ plants (Walker et al., 1999, 2001; Lea et al., 2001; Taylor et al., 2010). As decarboxylating enzyme, PCK is found in bundle sheath cells of C_4_ plants, where it plays a role in decarboxylating C_4_ acids (Walker and Leegood, 1996). Interestingly, there is support for evidence that PCK plays a role in decarboxylating C_4_ acids via a partial C_4_ cycle in the vascular system of some C_3_ plants (Hibberd and Quick, 2002).

Additionally, there are a few function-specific differences between C_4_ photosynthetic genes and their homologous genes in C_3_ and C_4_ plants, which are mostly governed by regulatory properties of gene specific expression (Sheen, 1999). Recent studies have confirmed that C_4_-*CA*, C_4_-*PEPC*, and C_4_-*PPDK* are localized in mesophyll cells, whereas C_4_-*NAD(P)-ME*, C_4_-*PCK*, and Rubisco (*RbcS*) are expressed at high levels in bundle sheath cells of leaves of the C_4_ plant maize (Langdale, 2011). Several studies in other species have identified the regulatory mechanisms controlling the expression patterns of these C_4_ photosynthetic enzymes (Offermann et al., 2006, 2008; Heimann et al., 2013). In plants, the acetylation of histone H3 lysine residue 9 (H3K9ac) and H4 lysine residue 5 (H4K5ac) is associated with gene transcriptional activation and is considered a marker of euchromatin (Lee et al., 2010; Heimann et al., 2013; Hou et al., 2015; Wang et al., 2015). Current evidence suggests that increases in the level of H3K9ac and H4K5ac in the promoters of C_4_ photosynthetic enzyme genes upon illumination are linked to transcriptional activation (Heimann et al., 2013). A recent study showed that *C_4_-PPDK*, *C_4_-PCK*, and their non-C_4_ homologous genes have organ-specific expression patterns, and in maize the level of H3K9ac and H4K5ac modifications correlates well with the mRNA level of most *PPDK* and *PCK* genes (Dong et al., 2016). Given that genes encoding C_4_ photosynthetic enzyme evolved from their C_3_ counterparts, it is hypothesized that similar regulatory mechanisms may underlie the regulation of these homologous gene expressions in C_3_ plants.

To assess the relationship between homologous C_4_ photosynthetic enzyme gene expression and histone acetylation modification in C_3_ woody plants, we cloned nine C_4_ photosynthetic enzyme gene homologs from the hybrid poplar *Populus simonii* × *Populus nigra*. We investigated their expression patterns as well as the enrichment of H3K9ac and H4K5ac modifications in the promoters of these genes in leaves, stem chlorenchyma (Sc), stem vascular tissue (Sv) and roots. Overall, our results show that *PsnCAs*, *PsnPPDKs*, *PsnPCKs*, and *PsnPEPCs* have tissue-dependent expression patterns, and most of these genes’ transcript abundances are well-correlated with the levels of H3K9ac and H4K5ac. Our results and previous research on the C_3_ herbaceous plant *Arabidopsis thaliana* (Zhou et al., 2010) and the C_4_ gramineous plants sorghum and maize (Heimann et al., 2013) suggest that the regulation of the expression of C_4_ photosynthetic genes and their C_3_ homologs by histone acetylation is conserved. Based on this similar regulatory mechanism it may be possible to modify C_3_ photosynthetic gene expression to be more C_4_-like as a part of future efforts to engineer C_3_ plants that are capable of C_4_ photosynthesis.

## Materials and Methods

### Plant Materials and Treatment

**FIGURE 1:**
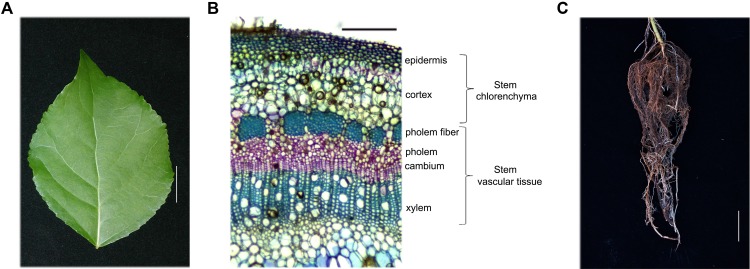
Hybrid poplar tissue and cell types used in this study. **(A–C)** Tissues collected from 5-month-old *P. simonii* × *P. nigra* grown in a greenhouse. **(A)** Leaf morphology. Scale bar, 2 cm. **(B)** Cross-section of a stem stained with toluidine blue. The cell types are labeled on the right. Many chloroplasts can be seen in the epidermis and cortex cells, which are called the stem chlorenchyma in our study. Phloem fiber, phloem, cambium and xylem cells are called stem vascular tissue. Scale bar, 200 μm. **(C)** Root morphology. Scale bar, 5 cm.

The poplar hybrid *P. simonii* × *P. nigra* (hereafter referred to as poplar) was used for all studies. Poplar seedlings were grown for 1 month under a 16 h day/8 h night cycle at 25°C in a tissue culture vessel containing MH medium, pH 5.8, plus 2% sucrose and 0.7% agar. Subsequently, healthy poplar seedlings were transferred to a mixture of soil and vermiculite (1:1) and cultivated in a greenhouse under a 16 h day (25°C)/8 h night (22°C) cycle for 5 months. Leaves from the fourth internode to the sixteenth internode, stem chlorenchyma (Sc) and stem vascular tissue (Sv) from the sixth internode to the eighteenth internode, and root tissues were harvested from 20 independent 5-month-old poplar plants 4 h after the onset of illumination (**Figure 1**).

Twelve additional 5-month-old poplar plants grown in a greenhouse were sprayed with a low concentration (2.5 and 5 μM) of TSA (Sigma, St. Louis, MO, United States) for 2 days to study the correlation between histone acetylation and gene expression. All tissues were immediately frozen in liquid N_2_ and completely ground using the 6870 Freezer Mill (SPEX SamplePrep, Metuchen, NJ, United States).

### Microscopic Observation

Free-hand cross-sections, 25–30 μm thick, of the 12th internode of fresh stems were stained with 0.1% toluidine blue (Sigma, St. Louis, MO, United States). Sections were observed under an OLYMPUS microscope (OLYMPUS, Japan) equipped with a computer-assisted digital camera MODEL ARTCAM-1400MI-WOM (ARTRAY, Japan).

### Chlorophyll Content Measurement

Chlorophyll content was measured using a previously published method (Ni et al., 2009). 300 mg of leaf, Sc and Sv tissues from 5-month-old poplar were ground in a mortar and pestle under liquid nitrogen. The ground tissue was transferred to 15 ml Falcon tubes, 5 ml 80% acetone was added, and samples were then mixed in the dark for 30 min. Subsequently, the tubes were centrifuged at 3,000 rpm at 4°C for 15 min, and the supernatant was transferred to a new tube. These procedures were repeated twice. A spectrophotometer (MAPADA, Shanghai, China) was used to measure to the absorbance due to chlorophyll at 663 and 645 nm. Chlorophyll content was determined for at least six independent samples per tissue.

### RNA Extraction, DNase I Digestion, and cDNA Synthesis

Total RNA was extracted from 100 mg of powdered leaves, Sc, Sv, and roots of poplar using the pBIOZOL Plant Total RNA Extraction Reagent according to the manufacturer’s instructions (BioFlux, Tokyo, Japan). The concentration of RNA was determined using the Nanodrop 2000 (Thermo Fisher, Waltham, MA, United States), and the sample was resolved on a 1.2% agarose gel to check the integrity of the RNA. To remove genomic DNA contamination, 1 μg of RNA was digested using the DNase I digestion Reagent Kit (Invitrogen, Carlsbad, CA, United States). Total cDNA was synthesized using the M-MLV Reverse Transcriptase Reagent Kit (Invitrogen, Carlsbad, CA, United States) and an oligo(dT) primer. A working sample of cDNA was prepared by diluting five-fold with sterile water and storing at -20°C.

### Gene Cloning and Vector Construction

The genomic sequences of poplar genes homologous to maize C_4_ photosynthesis enzyme genes *CA*, *PPDK*, *PCK*, and *PEPC* were obtained from the poplar sequence database^1^ (Supplemental Table S1). Full-length sequences of these genes were amplified from cDNA synthesized from poplar leaf or root RNA with KOD polymerase (NEB) according to the manufacture’s protocol using the primers listed in Supplemental Table S2. PCR products were purified with the TIANgel mini purification kit (TIAN GEN, Beijing, China), cloned into the pEasy-blunt cloning vector (Trans Gene, Beijing, China), and sequenced. The upstream sequences (about 2 kb in length) corresponding to the promoters of these genes were amplified from poplar genomic DNA using the primers listed in Supplemental Table S3.

### Selection of Reference Genes

Quantitative real-time polymerase chain reaction is a reliable technique for quantifying gene expression, and requires stable reference genes for data normalization (Xiao et al., 2015). However, no single reference gene has stable expression under all experimental conditions (Podevin et al., 2012). Therefore, it is necessary to choose a suitable reference gene which is expressed at a relatively stable level across the conditions being tested.

In our study, five housekeeping genes, *ACTIN2*, *TUBLIN*, *UBIQUITIN* (*UBQ*), *Elongation Factor 1a* (*EF1a*), and *18S ribosomal RNA* (*18S rRNA*) were tested for suitability as reference genes for gene expression studies in four vegetative tissues, including leaves, Sc, Sv, and roots, as well as in TSA-treated *P. simonii* × *P. nigra*. These gene sequences were obtained from the *Populus trichocarpa* genome annotation v3.0^2^. QRT-PCR was performed using the primers listed in Supplemental Table S4 to evaluate the expression variation of these candidates across tissues. The average cycle threshold (*C*q) values of candidate genes ranged from 10.68 to 22.76 in the four tissues (Supplemental Figure S2A). Except for *18S* rRNA, all genes showed low variability in *C*q value between leaves, Sc, Sv, and roots.

The average expression stability value (*M*-value) is a parameter used by the geNorm software program to identify the best reference genes. The lower the *M*-value is, the more stable the gene expression (Bustin et al., 2009). We also used geNorm software to select the most stable reference genes (Dong et al., 2016). Based on geNorm analysis, *ACTIN2* and *EF1a* were the most stable genes among the five candidate reference genes (Supplemental Figure S2B). In a previous study, *PtACTIN2* was used as reference gene for diverse tissues of 1-year-old *P. trichocarpa*, including differentiated or mature xylem and phloem (Yu et al., 2013). Therefore, we chose to use *ACTIN2* as a reference gene in our study.

### Chromatin Immunoprecipitation (ChIP)

We carried out ChIP as previously described (Bowler et al., 2004; Gendrel et al., 2005; Li et al., 2014) with the following modifications: 1.5 g of leaves, Sc, Sv, and roots from 5-month-old poplar plants grown in a greenhouse were harvested separately and cross-linked with 1% (wt/wol) formaldehyde for 15 min at 4°C. The purified chromatin was sheared to 0.3–0.7 kb fragments by sonicating under cooling for 4 min (4 s-on, 10 s-off) at 15% amplitude using a Vibra-cell VCX-505 sonicator (Sonics, Newtown, CT, United States). The sheared chromatin was diluted with ChIP dilution buffer and pre-cleared with 100 μl of protein A agarose (Millipore, Billerica, MA, United States) (Bowler et al., 2004; Gendrel et al., 2005).

For each sample, 1 ml of diluted pre-cleared chromatin was used for immunoprecipitation, and a 10 μl aliquot was used to quantify the amount of input. To immunoprecipitate chromatin containing histone modifications, 100 μl of protein A agarose and 5 μl anti-acetyl H3K9 (07-352, Millipore, Billerica, MA, United States) or 5 μl anti-acetyl H4K5 (07-327, Millipore, Billerica, MA, United States) were added to the diluted pre-cleared chromatin. Inmunocomplexes were washed with buffer in the following order: salt buffer, LiCl buffer, and TE buffer (Li et al., 2014). After discarding TE buffer, the immunoprecipitated chromatin was eluted with elution buffer (Li et al., 2014). After formaldehyde cross-linking, ChIP-DNA was purified by phenol/chloroform/isoamyl alcohol extraction followed by ethanol precipitation (Li et al., 2014).

### Quantitative Real-Time Polymerase Chain Reaction (QRT-PCR)

Quantitative real-time polymerase chain reaction was performed to profile the expression patterns of reference genes, poplar homologs of C_4_ photosynthetic enzyme genes and the changes in expression of these genes in TSA-treated poplar. Specific primers were designed using the online Integrated DNA Technologies software^3^ and are listed in Supplemental Tables S4, S5. QRT-PCR reactions were performed using SYBR *Premix Ex Taq* (Takara, Shiga, Japan) on a LightCycler 480 system (Roche, Basel, Sweden). Two microliters of diluted cDNA sample was used as template in an amplification reaction volume of 10 μl. The amplification program consisted of 30 s of initial denaturation at 95°C, followed by 40 cycles of 10 s at 95°C, 20 s at 60°C and 20 s at 72°C, and ended with a final extension step at 72°C for 20 s. Three replicated reactions per sample were done. For the analysis of the expression of poplar homologs of C_4_ photosynthetic enzyme genes, all samples were normalized to the reference gene *PsnACTIN2*. The final relative expression was calculated using the formula *F* = 2^-ΔΔ*Ct*^ (Chen et al., 2014).

For ChIP assays, 2 μl ChIP-DNA sample was used as template in an amplification reaction volume of 10 μl. Primer positions and sequences are listed in Supplemental Figures S3, S4 and Table S6. The amplification program consisted of 30 s of initial denaturation at 95°C, followed by 45 cycles of 10 s at 95°C, 20 s at 60°C and 20 s at 72°C. Relative enrichment of H3K9ac and H4K5ac in the promoters of the *PsnCA*, *PsnPPDK*, *PsnPCK*, and *PsnPEPC* genes was calculated by normalizing to the value of these marks in the promoter of the *PsnACTIN2* housekeeping gene. Relative enrichment values are based on the average of three PCR reactions for each sample.

### Western Blotting

Total proteins from leaves, Sc, Sv, and roots of control and TSA-treated poplar were extracted using a phenol (Sigma, St. Louis, MO, United States) extraction procedure as described previously (Hurkman and Tanaka, 1986; Wang et al., 2011). Protein concentrations were measured using the Bio-Rad Protein Assay (Bio-Rad, United States). Thirty micrograms of protein was separated by 15% SDS-PAGE gel and transferred by electroblotting (180 mA for 3 h) to a PVDF membrane (Millipore, Billerica, MA, United States). Membranes were blocked with 5% skim milk and probed with anti-H3K9ac (07-352, Millipore, Billerica, MA, United States) and anti-H4K5ac (07-327, Millipore, Billerica, MA, United States) antibodies to detect histone marks. Polyclonal anti-actin (EASYBIO, Beijing, China) antibody was used as a control for protein loading. Fluorescence goat anti-rabbit (GAR) antibody (Odyssey, United States) was used as the secondary antibody. Membranes were digitized using a two-photo far infrared scanner (Odyssey, United States) and analyzed with Image Studio software^4^. Western blotting experiments were repeated three times for each protein sample.

## Results

### Isolation of Poplar Genes Homologous to C_4_ Photosynthetic Enzyme Genes

The *P. simonii* × *P. nigra* genome has not been sequenced. Therefore, we used the known protein sequences of maize C_4_-CA (GRMZM2G121878), C_4_-PPDK (GRMZM2G306345), C_4_-PCK (GRMZM2G001696), and C_4_-PEPC (GRMZM2G083841) as queries to blast against the *P. trichocarpa* genome database^1^. Using primers specific to the *P. trichocarpa* sequences and cDNA from *P. simonii* × *P. nigra* leaves or roots, we cloned nine *P. simonii* × *P. nigra* genes homologous to the maize *C_4_-CA*, *C_4_-PPDK*, *C_4_-PCK*, and *C_4_-PEPC* genes (Supplemental Table S1). Sequence alignments showed that the PsnCA1 and PsnCA2 proteins shares 91.6% identity, and both are different from PsnCA3. Compared with PsnCA3, the N-terminal ends of both the PsnCA1 and PsnCA2 proteins are shorter by 76 amino acids and both C-terminal ends are 8 amino acids shorter (Supplemental Figure S1A). We cloned two transcripts (named *PsnPPDK1-1* and *PsnPPDK1-2*) for a single *PsnPPDK* gene, and the protein sequences share 88.08% identity (Supplemental Figure S1B). Note that these transcripts are transcribed from different promoters; therefore, for the purposes of our analysis, we treat them as independently transcribed genes. There are two *PsnPCK* genes, the protein sequences of which share 82.76% identity (Supplemental Figure S1C). In addition, we also identified two *PsnPEPC* genes, the proteins sequences of which share 94.32% identity (Supplemental Figure S1D).

### *PsnCA*, *PsnPPDK*, *PsnPCK*, and *PsnPEPC* Genes Have Tissue-Dependent Expression Patterns

**FIGURE 2:**
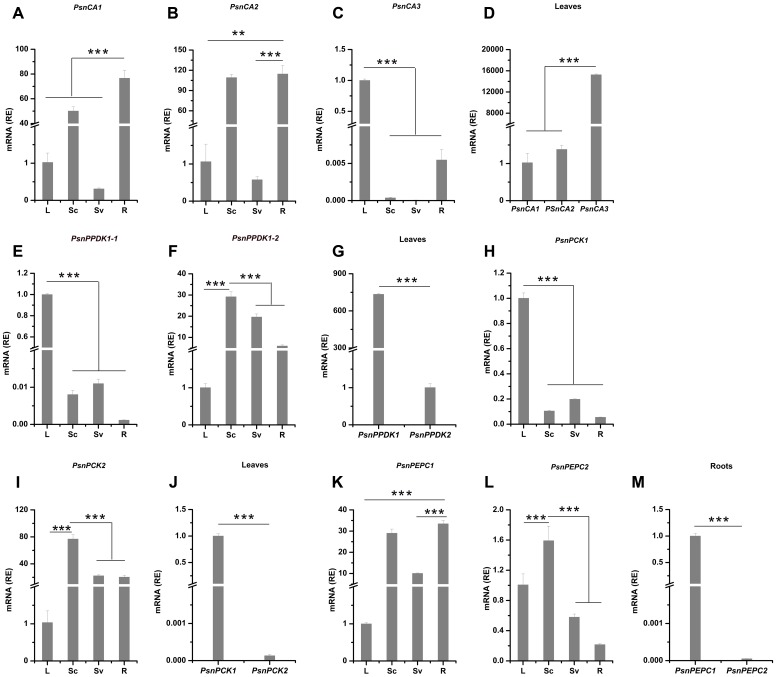
The relative expression levels of poplar genes homologous to C_4_ photosynthetic enzyme genes in different tissues of poplar. **(A–M)** All tissues were harvested from 5-month-old poplar plants grown in a greenhouse 4 h after the onset of illumination. The expression levels of *PsnCA* genes **(A–D)**, *PsnPPDK* genes **(E–G)**, *PsnPCK* genes **(H–J)** and *PsnPEPC* genes **(K–M)** genes. Expression levels were normalized to *PsnACTIN2* expression. All relative enrichment (RE) values are means of at least three independent experiments. Bars indicate SE. Leaves (L), stem chlorenchyma (Sc), stem vascular tissue (Sv), and roots (R). Asterisks indicated significantly different means (^∗∗∗^*p* < 0.001) as determined with a *t*-test.

To better understand the potential functions of poplar genes homologous to C_4_ photosynthetic enzyme genes, we profiled their expression patterns in leaves, Sc (includes epidermis and cortex), Sv (includes phloem fiber, phloem, cambium and xylem), and roots (**Figures 1**, **2**). Based on the average cycle threshold (*C*q) value and the average expression stability value (*M*-value), we chose to use *ACTIN2* as a reference gene in this study (Supplemental Figure S2). Among the *CA* genes, both *PsnCA1* and *PsnCA2* were highly expressed in roots and Sc (**Figures 2A,B**). However, *PsnCA3* was most highly expressed in leaves (**Figure 2C**). Surprisingly, the level of *PsnCA3* transcript was nearly 15,000-fold higher than *PsnCA1* and *PsnCA2* in leaves (**Figure 2D**). The *PPDK* and *PCK* gene pairs showed similar differences in expression pattern. For example, both *PsnPPDK1-1* and *PsnPCK1* were highly expressed in leaves, whereas *PsnPPDK1-2* and *PsnPCK2* were highly expressed in Sc and Sv (**Figures 2E–J**). Unlike the *CA*, *PPDK*, and *PCK* genes, neither *PEPC* gene was most highly expressed in leaves. The abundance of both transcripts was high in Sc, but the expression level of *PsnPEPC1* in roots was much higher than *PsnPEPC2*(**Figures 2K–M**).

Based on the presence or absence of tissue chlorophyll (**Figure 1** and Supplemental Figure S3) and gene-specific expression patterns (**Figure 2**), we generally classified these nine genes into two clusters. Genes in one cluster, including *PsnCA3*, *PsnPPDK1-1*, *PsnPPDK1-2*, *PsnPCK1*, *PsnPCK2*, and *PsnPEPC2*, are highly expressed in photosynthetic tissues (chlorophyll content in the leaves and Sc is 1.42 and 0.32 mg/g, respectively). Genes in the second cluster, including *PsnCA1*, *PsnCA2*, and *PsnPEPC1*, are highly expressed in non-photosynthetic tissues (chlorophyll content in Sv and roots is 0.002 and 0 mg/g).

### The Levels of H3K9ac and H4K5ac in the Promoters of the *PsnCA*, *PsnPPDK*, *PsnPCK*, and *PsnPEPC* Genes Correlate Well with Tissue-Dependent Expression Patterns

Previous studies have found that for many plant loci, histone modification within the promoter and gene body is involved in the tissue-specific regulation of gene expression (Cai et al., 2003; Heintzman et al., 2009; Zhou et al., 2010; Ong and Corces, 2011; Heimann et al., 2013), and histone acetylation has been extensively correlated with transcriptional activation (Wolffe and Hayes, 1999; Turner, 2000). Therefore, we asked whether histone acetylation modification is correlated with the tissue-dependent expression of the *PsnCA*, *PsnPPDK*, *PsnPCK*, and *PsnPEPC* genes. We analyzed the levels of H3K9ac and H4K5ac marks in the promoters of these genes using a ChIP assay. Promoter sequences upstream of the transcription initiation site (TIS) of these genes (∼2 kb) were divided into three regions, including the distal region (P1), middle region (P2) and proximal region (P3) (Supplemental Figure S4). Immunoprecipitation efficiency of the *PsnACTIN2* gene promoter was used to correct for variation in the amount of chromatin prepared from leaves, Sc, Sv, and roots (Supplemental Figure S5).

**FIGURE 3:**
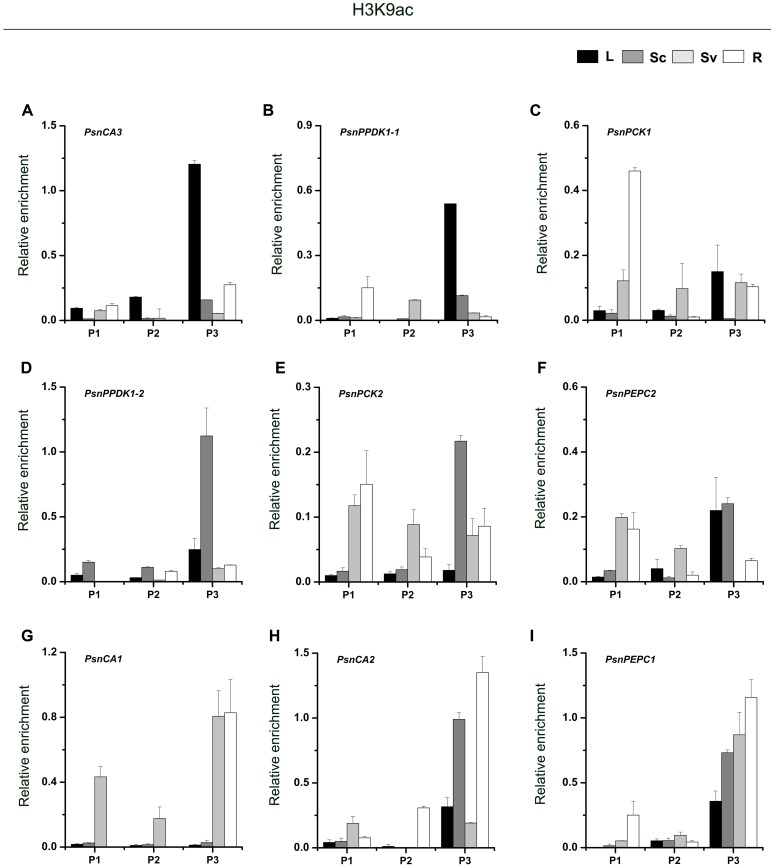
ChIP analysis of the *PsnCA*, *PsnPPDK*, *PsnPCK*, and *PsnPEPC* gene promoters using the H3K9ac antibody. **(A–I)** All tissues were harvested from 5-month-old poplar plants grown in a greenhouse 4 h after the onset of illumination. ChIP was used to detect H3K9ac levels in the upstream promoters of the *PsnCA*, *PsnPPDK*, *PsnPCK*, and *PsnPEPC* genes in leaves (L), stem chlorenchyma (Sc), stem vascular tissue (Sv), and roots (R). P1, P2, and P3 represent the distal, middle, and proximal promoter regions (Supplemental Figure S4). Levels of H3K9ac were standardized by the accumulation of H3K9ac in the promoter of the *PsnACTIN2* gene (Supplemental Figure S5). Values are the means from three independent experiments. Bars indicate SE.

As shown in **Figure 3**, for almost genes, the level of H3K9ac in the P3 region (close to the TIS) was higher than in the P1 and P2 regions. In photosynthetic tissues, the highest enrichment of H3K9ac in the P3 region of *PsnCA3*, *PsnPPDK1-1*, and *PsnPCK1* was detected in leaves (**Figures 3A–C**). This correlates well with the greater transcript abundance of these genes in leaves (**Figures 2C,E,H**). In Sc, relatively high enrichment of H3K9ac was observed in the promoters of the *PsnPPDK1-2*, *PsnPCK2*, and *PsnPEPC2* genes, which are highly expressed in this tissue (**Figures 3D–F**). In the non-photosynthetic root tissue, we found that H3K9ac was strongly enriched in the promoters of *PsnCA1*, *PsnCA2*, and *PsnPEPC1* which correlates with the high level of expression of these genes in roots (**Figures 3G–I**). We also found enrichment of H3K9ac in the P1 and P2 regions of these genes, but there was no obvious regular changes in both two regions between tissues (**Figures 3A–I**).

**FIGURE 4:**
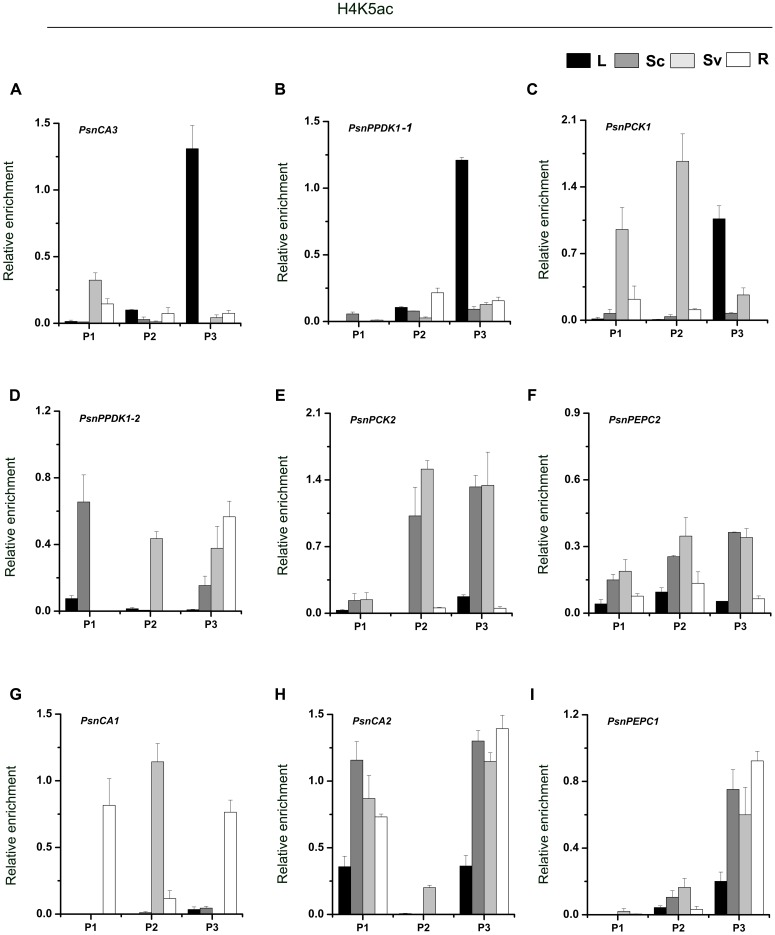
ChIP analysis of the *PsnCA*, *PsnPPDK*, *PsnPCK*, and *PsnPEPC* gene promoters using the H4K5ac antibody. **(A–I)** All tissues were harvested from 5-month-old poplar plants grown in a greenhouse 4 h after the onset of illumination. ChIP was used to detect H3K9ac levels in the upstream promoters of the *PsnCA*, *PsnPPDK*, *PsnPCK*, and *PsnPEPC* genes in leaves (L), stem chlorenchyma (Sc), stem vascular tissue (Sv), and roots (R). P1, P2, and P3 represent the distal, middle, and proximal promoter regions (Supplemental Figure S4). Levels of H3K9ac were standardized by the accumulation of H3K9ac in the promoter of the *PsnACTIN2* gene (Supplemental Figure S5). Values are the means from three independent experiments. Bars indicate SE.

As shown in **Figure 4**, we found high levels of H4K5ac in the P3 region of almost target genes, which is similar to the levels of H3K9ac in that region. In terms of H4K5ac modification, we also found that strong enrichment in the P3 region of most genes was correlated with high transcript accumulation. For example, H4K5ac was highly enriched in the P3 region of the *PsnCA3*, *PsnPPDK1-1*, and *PsnPCK1* genes in leaves (**Figures 4A–C**), and in the *PsnCA1*, *PsnCA2*, and *PsnPEPC1* genes in roots (**Figures 4G–I**). For the *PsnPCK2* and *PsnPEPC2* genes, which are highly expressed in the photosynthetic tissue Sc, strong enrichment of H4K5ac in the P3 region was observed in both Sc and Sv (**Figures 4E,F**). However, we found the highest enrichment of H4K5ac in the P1 region of the *PsnPPDK1-2* gene in Sc, not in the P3 region (**Figure 4D**).

The correlation between H3K9ac and H4K5ac levels and expression levels of the *PsnCA*, *PsnPPDK*, *PsnPCK*, and *PsnPEPC* genes indicates that histone acetylation modification may regulate the tissue-dependent expression of genes homologous to C_4_ photosynthetic enzyme genes in C_3_ woody plants.

### Application of Exogenous HDAC Inhibitors Alters Histone Acetylation and the Expression of *PsnCA*, *PsnPPDK*, *PsnPCK*, and *PsnPEPC* Genes

**FIGURE 5:**
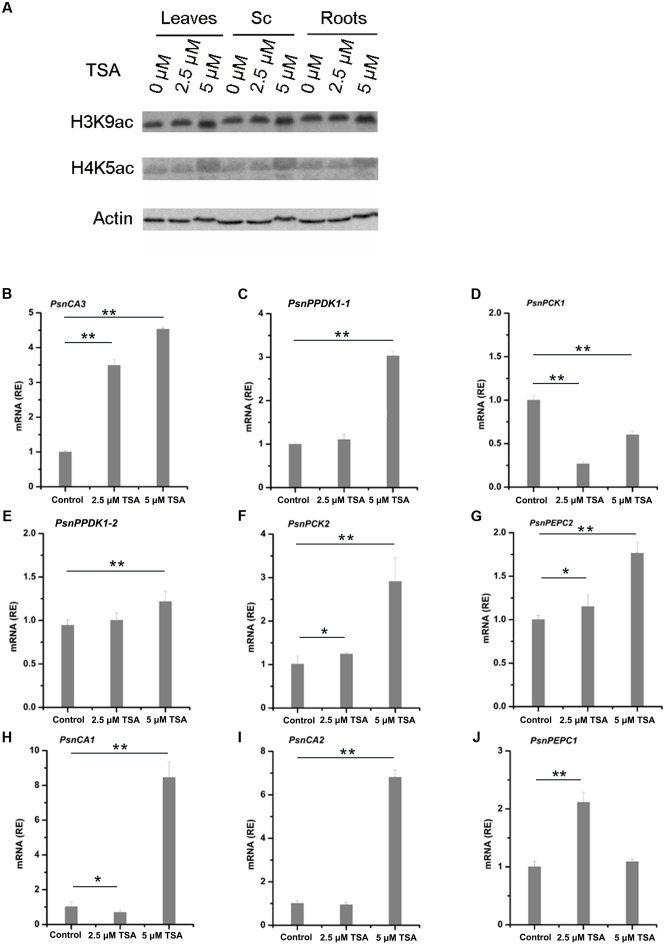
TSA affects H3K9ac and H4K5ac protein levels and mRNA levels of poplar homologs of C_4_ photosynthetic enzyme genes. **(A)** Western blot analysis of H3K9ac and H4K5ac protein levels in leaves (L), stem chlorenchyma (Sc), and roots (R) of poplar treated with TSA for 2 days. ACTIN was used as a control for equal loading. Expression of the *PsnCA3*, *PsnPPDK1-1*, and *PsnPCK1* genes in leaves **(B–D)**, the *PsnPPDK1-2*, *PsnPCK2*, and *PsnPEPC2* genes in Sc **(E–G)**, and the *PsnCA1*, *PsnCA2*, and *PsnPEPC1* genes in roots **(H–J)** of poplar treated with TSA for 2 days. All expression levels were normalized to *PsnACTIN2* expression. Values are means from three independent experiments. Bars indicate SE. Asterisks indicated significantly different means (^∗^*p* < 0.05; ^∗∗^*p* < 0.005) as determined with a *t*-test.

Treatment with the HDAC inhibitor, TSA, results in the accumulation of acetylated histones in the genome (Bernstein et al., 2000). In order to further study the relationship between histone acetylation and tissue-dependent expression of the *PsnCA*, *PsnPPDK*, *PsnPCK*, and *PsnPEPC* genes, we applied low concentrations (2.5 and 5 μM) of TSA to 5-month-old poplar plants and measured levels of histone acetylation and gene expression in leaves, Sc, Sv and roots (Supplemental Figures S6, S7). As we expected, western blot analysis showed that application of TSA induced a slight increase in both H3K9ac and H4K5ac in leaves, Sc, Sv, and roots (**Figure 5A** and Supplemental Figure S7A). Moreover, qRT-PCR results showed that TSA altered the level of gene expression to different extents in different tissues (Supplemental Figures S7B–J). In general, TSA significantly increased the abundance of *PsnCA3* and *PsnPPDK1-1* transcripts in leaves (**Figures 5B,C**), *PsnPPDK1-2*, *PsnPCK2*, and *PsnPEPC2* transcripts in Sc (**Figures 5E–G**), and *PsnCA1*, *PsnCA2*, and *PsnPEPC1* transcripts in roots (**Figures 5H–J**) compared to untreated tissues. However, application of TSA significantly reduced the transcript level of *PsnPCK1* in leaves (**Figure 5D**), suggesting that TSA probably has pleiotropic effects on other regulatory networks linked to this gene.

### Enrichment of H3K9ac and H4K5ac Is Strongly Correlated with the Level of Tissue-Dependent Expression of the Poplar *PsnCA*, *PsnPPDK*, *PsnPCK2*, and *PsnPEPCs* Genes

**FIGURE 6:**
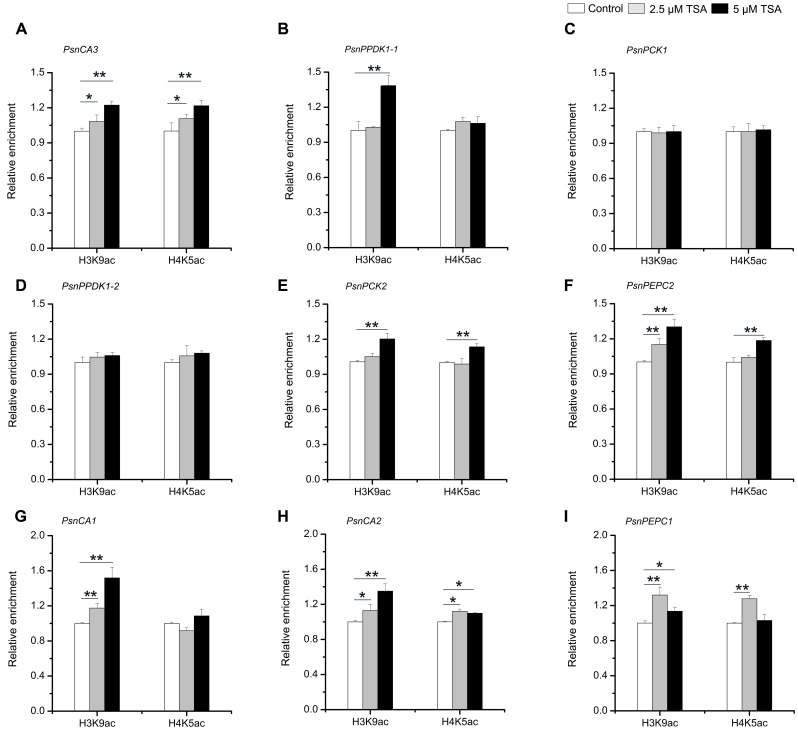
TSA affects the level of H3K9ac and H4K5ac in the promoters of the poplar *PsnCA*, *PsnPPDK*, *PsnPCK*, and *PsnPEPC* genes. ChIP was used to detect H3K9ac and H4K5ac levels in the P3 (close to TIS) promoter region of the *PsnCA3*, *PsnPPDK1-1*, and *PsnPCK1* genes in leaves (L) **(A–C)**, the *PsnPPDK1-2*, *PsnPCK2*, and *PsnPEPC2* genes in stem chlorenchyma (Sc) **(D–F)**, and the *PsnCA1*, *PsnCA2*, and *PsnPEPC1* genes in roots (R) **(G–I)** of poplar treated with TSA for 2 days. Levels of H3K9ac and H4K5ac were standardized by the accumulation of H3K9ac and H4K5ac in the promoter of the *PsnACTIN2* gene (Supplemental Figure S4). Values are the means from three independent experiments. Bars indicate SE. Asterisks indicated significantly different means (^∗^*p* < 0.05; ^∗∗^*p* < 0.005) as determined with a *t*-test.

In order to further confirm that H3K9ac and H4K5ac regulate the tissue-dependent expression patterns of the *PsnCA*, *PsnPPDK*, *PsnPCK*, and *PsnPEPC* genes, we performed ChIP assays to detect the enrichment of H3K9ac and H4K5ac in the promoters of these genes in TSA-treated poplar. The accumulation of H3K9ac and H4K5ac was altered by TSA to different extents in the P3 regions of almost all genes detected in different tissues (Supplemental Figures S8, S9). In general, the enrichment of both H3K9ac and H4K5ac in the P3 regions of *PsnCA3* in leaves, *PsnPPDK1-2*, *PsnPCK2* and *PsnPEPC2* in Sc, as well as *PsnCA2* and *PsnPEPC1* in roots was enhanced to different degrees (**Figures 6A,D,E,F,H,I**). Additionally, the enrichment of H3K9ac in the P3 regions of *PsnPPDK1-1* in leaves and *PsnCA1* in roots was significantly induced by TSA (**Figures 6B,G**). Moreover, the levels of H3K9ac and H4K5ac were in agreement with the transcript levels observed for these genes under TSA treatment (**Figure 5**). In contrast, no obvious change in H3K9ac or H4K5ac level was detected for *PsnPCK1* (**Figure 6C**), indicating that in leaves H3K9ac and H4K5ac do not play important roles in the regulation of *PsnPCK1* gene expression. Taken together, these results reveal that histone acetylation modification is correlated with the tissue-dependent expression of most poplars homologs of C_4_ photosynthesis genes.

## Discussion

Analysis of genome sequences for a growing number of species has indicated that all of the enzymes required for C_4_ photosynthesis (such as CA, PPDK, PCK, and PEPC) are present in C_3_ plants, and even in algae and microorganisms. Moreover, C_3_ homologs of C_4_ photosynthetic enzyme genes share high sequence identity with their C_4_ counterparts. During the evolution from C_3_ to C_4_ plants, photosynthetic genes acquired new regulatory features, such as cell type-specific expression in mesophyll or bundle sheath cells. It is likely that duplication of genes encoding C_4_ photosynthesis proteins allowed ancestral functions to be maintained in one duplicate copy, while also permitting neofunctionalization of the other copy, leading to C_4_-specific roles and expression patterns (Ludwig, 2013).

Much of the regulation of C_4_ photosynthetic enzyme expression takes place at the promoter (Sheen, 1999; Taniguchi et al., 2000; Hibberd and Covshoff, 2010), and increasing evidence indicates that histone modification plays an important role in the regulation of tissue-specific expression of *CA*, *PPDK*, *PCK*, *PEPC*, and *RbcS2* in C_4_ plants (Offermann et al., 2008; Heimann et al., 2013; Dong et al., 2016). Except for studies on the role of histone acetylation modifications in the regulation of the *RbcA* and *RbcS* genes during *Pinus radiate* needle development (Charron et al., 2009; Valledor et al., 2015), the role of histone modifications in the regulation of photosynthesis genes in C_3_ plants has not been well studied. Given that there is evidence that the C_4_ forms of photosynthetic enzymes have evolved from C_3_ counterparts, for example CA3 (β-CA in C_4_ plant *Flaveria bidentis*) and the C_4_ form of PPDK, evolved from chloroplastic C_3_ ancestors (Langdale, 2011; Ludwig, 2012), we hypothesized that histone modification plays a significant role in the regulation of photosynthetic genes expression in C_3_ plants. To test this hypothesis, we analyzed the relationship between histone acetylation modification and expression for poplar homologs of C_4_ photosynthetic genes.

We first isolated nine poplar homologs of the maize *C_4_-CA*, *C_4_-PPDK*, *C_4_-PCK*, and *C_4_-PEPC* genes and investigated the expression of these genes in photosynthetic and non-photosynthetic tissues. We found these nine poplar homologs of C_4_ genes had tissue-dependent expression pattern. For example, *PsnCA3*, *PsnPPDK1-1*, and *PsnPCK1* are highly expressed in photosynthetic tissue leaves (**Figure 2**), which is consistent with the reported expression patterns of their homologous genes in C_3_ herbaceous species and C_4_ gramineous species (Fett and Coleman, 1994; Malone et al., 2007; Taylor et al., 2010; Langdale, 2011; Dong et al., 2016). However, not all poplar photosynthetic gene expression patterns are conserved with their homologs in other species. In contrast to AtPEPC1 and AtPEPC2, which account for nearly 93% of the total PEPC activity in leaves (Shi et al., 2015), *PsnPEPC1* and *PsnPEPC2* was highly expressed in roots, and Sc, respectively (**Figure 2**).

Reviewed in DiMario’s paper, various studies have shown that plants have many genes encoding CA, PPDK, PCK, and PEPC, which are found in most tissues and many intracellular compartments. In addition to uncertain roles in photosynthesis, the functions of these homologous genes are also required for many metabolic, signaling, and developmental pathways in C_3_ species. There is evidence that C_3_ β-CAs function in carbon-concentration, nitrogen-fixation, stomatal movement and development, biotic and abiotic stress responses, and amino acid as well as lipid biosynthesis (DiMario et al., 2017). A single *PPDK* gene that possesses a dual promoter giving rise to two transcripts is found in many C_3_ herbaceous species in addition to poplar, including *Arabidopsis*, wheat, rice, *Flaveria*, and even in the C_4_ species maize. In all cases the longer *PPDK* transcript (homologous to *PsnPPDK1-1*) encodes a protein that is targeted to the chloroplast of leaves, and the smaller protein (homologous to PsnPPDK1-2) is cytosolic (Aoyagi and Bassham, 1984; Glackin and Grula, 1990; Rosche and Westhoff, 1995; Imaizumi et al., 1997; Parsley and Hibberd, 2006). PCK is localized in phloem and trichome tissues, oil and resin ducts, developing seeds, and ripening tomato fruit (Leegood and Walker, 1999; Walker et al., 1999; Bahrami et al., 2001), suggesting it has various roles in plant development, such as gluconeogenesis, nitrogen, and amino acid metabolism. Our finding that the *PsnPEPC2* gene is highly expressed in Sc is consistent with the finding that in woody plants PEPC enzyme activity is higher in current-year stems and also with PEPC’s function in sustaining the carbon flux (Berveiller et al., 2007). High expression of PEPC in the stem was possibly selected for because in addition to leaves, a strong stem is another major source of assimilated carbon in woody plants. Similar to what has been observed in some reported C_4_ plants, the poplar C_4_ genes homologs have similar expression patterns in that closely related genes, which likely have divergent roles in plant development, such as carbon and nitrogen metabolism.

We next asked whether the tissue-dependent expression patterns of the poplar *CA*, *PPDK*, *PCK*, and *PEPC* genes were correlated with histone acetylation modification. Our finding that for almost all genes, the peak of H3K9ac and H4K5ac accumulation was highest in the P3 region close to the TIS (**Figures 3**, **4**) suggests that the nucleosomes adjacent to the TIS of active genes are hypoacetylated compared to the surrounding regions. Similar observations were also made in *Arabidopsis* and Maize, where peaks of H3K9ac and H4K5ac accumulation were found around the ATG position, especially in photosynthesis genes (Zhou et al., 2010; Perduns et al., 2015; Dong et al., 2016). In the C_4_ plant maize, *PPDK* and *PCK* gene expression is regulated by histone modifications (Dong et al., 2016). Our findings that H3K9ac and H4K5ac modifications correlate well with tissue-dependent expression of the *PsnCA*, *PsnPPDK*, *PsnPCK*, and *PsnPEPC* genes and that, with the exception of *PsnPCK1*, treatment with HDAC inhibitor, TSA led to tissue-specific increases in H3K9ac or H4K5ac that were correlated with mRNA levels provide evidence that these C_4_ homologous genes are also regulated by histone modification. The similarities between histone acetylation regulation of photosynthetic enzyme genes in C_3_ and C_4_ plants suggests that this regulatory system seems conserved in diverse C_3_ and C_4_ species. It also provides evidences that a preexisting epigenetic mechanism for promoter control was probably recruited during the evolution of C_4_ plants.

The continued deep sequencing of more and more closely related C_3_ and C_4_ species will allow the functions of C_4_ photosynthetic enzyme homologs to be analyzed in lineages of C_3_ species that are closely related to C_4_ species and give insight into how the regulation and functions of these genes changed during the transition from C_3_ to C_4_ photosynthesis. Understanding the mechanism behind the recruitment of photosynthetic genes into new biochemical pathways and identification of key factors controlling C_4_ gene expression will enable us to engineer “real-C_4_” plants from C_3_ plants in the future.

## Author Contributions

YL and B-CW conceived and designed this work. YL performed the gene cloning, qRT-PCR, Western blotting, and TSA treatment. YL and X-MD performed the ChIP experiment. FJ performed morphological and anatomic experiments. ZS and QC provided technical help and suggestions. YL and B-CW wrote the article.

## Conflict of Interest Statement

The authors declare that the research was conducted in the absence of any commercial or financial relationships that could be construed as a potential conflict of interest.

## Footnotes

1 http://www.phytozome.net/poplar

2 https://phytozome.jgi.doe.gov/pz/portal.html

3 http://www.idtdna.com/scitools/Applications/RealTimePCR/

4 https://www.licor.com/bio/products/software/image_studio/

## Abbreviations

CA: carbonic anhydrase
ChIP: chromatin immunoprecipitation
H3K9ac: acetylation of histone H3 lysine 9
H4K5ac: acetylation of histone H4 lysine 5
HDAC: histone deacetylase
NAD-ME: NAD malic enzyme
NADP-ME: NADP malic enzyme
PCK: phosphoenolpyruvate carboxykinase
PEPC: phosphoenolpyruvate carboxylase
PPDK: pyruvate orthophosphate dikinase
qRT-PCR: quantitative real-time polymerase chain reaction
Rubisco: ribulose 1,5-bisphosphate carboxylase/oxygenase
TSA: trichostatin A

## References

[B1] Aoyagi K. Bassham J. A. (1984). Pyruvate orthophosphate dikinase of C3 seeds leaves as compared to the enzyme from maize. *Plant Physiol.* 75 387–392. 10.1104/pp.75.2.387 16663632 PMC1066918

[B2] Bahrami A. R. Chen Z.-H. Walker R. P. Leegood R. C. Gray J. E. (2001). Ripening-related occurrence of phosphoenolpyruvate carboxykinase in tomato fruit. *Plant Mol. Biol.* 47 499–506. 10.1023/A:1011842828723 11669575

[B3] Bernstein B. E. Tong J. K. Schreiber S. L. (2000). Genomewide studies of histone deacetylase function in yeast. *Proc. Natl. Acad. Sci. U.S.A.* 97 13708–13713. 10.1073/pnas.250477697 11095743 PMC17640

[B4] Berveiller D. Vidal J. Degrouard J. Ambard-Bretteville F. Pierre J. N. Jaillard D. (2007). Tree stem phosphoenolpyruvate carboxylase (PEPC): lack of biochemical and localization evidence for a C4-like photosynthesis system. *New Phytol.* 176 775–781. 10.1111/j.1469-8137.2007.02283.x 17997763

[B5] Bowler C. Benvenuto G. Laflamme P. Molino D. Probst A. V. Tariq M. (2004). Chromatin techniques for plant cells. *Plant J.* 39 776–789. 10.1111/j.1365-313X.2004.02169.x 15315638

[B6] Bustin S. A. Benes V. Garson J. A. Hellemans J. Huggett J. Kubista M. (2009). The MIQE guidelines: minimum information for publication of quantitative real-time PCR experiments. *Clin. Chem.* 55 611–622. 10.1373/clinchem.2008.112797 19246619

[B7] Cai S. Han H.-J. Kohwi-Shigematsu T. (2003). Tissue-specific nuclear architecture and gene expession regulated by SATB1. *Nat. Genet.* 34 42–51. 10.1038/ng1146 12692553

[B8] Charron J.-B. F. He H. Elling A. A. Deng X. W. (2009). Dynamic landscapes of four histone modifications during deetiolation in *Arabidopsis*. *Plant Cell* 21 3732–3748. 10.1105/tpc.109.066845 20008096 PMC2814509

[B9] Chen H. Xu X. L. Li Y. P. Wu J. X. (2014). Characterization of heat shock protein 90, 70 and their transcriptional expression patterns on high temperature in adult of *Grapholita molesta* (Busck). *Insect Sci.* 21 439–448. 10.1111/1744-7917.12057 24006328

[B10] DiMario R. J. Clayton H. Mukherjee A. Ludwig M. Moroney J. V. (2017). Plant carbonic anhydrases: structures, locations, evolution, and physiological roles. *Mol. Plant* 10 30–46. 10.1016/j.molp.2016.09.001 27646307 PMC5226100

[B11] Dong X.-M. Li Y. Chao Q. Shen J. Gong X.-J. Zhao B.-G. (2016). Analysis of gene expression and histone modification between C_4_ and non-C_4_ homologous genes of PPDK and PCK in maize. *Photosynth. Res.* 129 71–83. 10.1007/s11120-016-0271-9 27161567

[B12] Eastmond P. J. Germain V. Lange P. R. Bryce J. H. Smith S. M. Graham I. A. (2000). Postgerminative growth and lipid catabolism in oilseeds lacking the glyoxylate cycle. *Proc. Natl. Acad. Sci. U.S.A.* 97 5669–5674. 10.1073/pnas.97.10.5669 10805817 PMC25886

[B13] Fett J. P. Coleman J. R. (1994). Characterization and expression of two cDNAs encoding carbonic anhydrase in *Arabidopsis thaliana*. *Plant Physiol.* 105 707–713. 10.1104/pp.105.2.707 7520589 PMC159412

[B14] Gendrel A.-V. Lippman Z. Martienssen R. Colot V. (2005). Profiling histone modification patterns in plants using genomic tiling microarrays. *Nat. Methods* 2 213–218. 10.1038/nmeth0305-213 16163802

[B15] Glackin C. A. Grula J. W. (1990). Organ-specific transcripts of different size and abundance derive from the same pyruvate, orthophosphate dikinase gene in maize. *Proc. Natl. Acad. Sci. U.S.A.* 87 3004–3008. 10.1073/pnas.87.8.3004 2158100 PMC53822

[B16] Gutierrez M. Huber S. Ku S. Kanai R. Edwards G. (1974). “Intracellular localization of carbon metabolism in mesophyll cells of C_4_ plants,” in *Proceedings of the Third International Congress on Photosynthesis*, (Amsterdam: Elsevier Science Publishers), 1219–1230.

[B17] Hatch M. Kagawa T. Craig S. (1975). Subdivision of C_4_-pathway species based on differing C_4_ acid decarboxylating systems and ultrastructural features. *Funct. Plant Biol.* 2 111–128. 10.1071/PP9750111

[B18] Hatch M. D. Burnell J. N. (1990). Carbonic anhydrase activity in leaves and its role in the first step of C_4_ photosynthesis. *Plant Physiol.* 93 825–828. 10.1104/pp.93.2.825 16667544 PMC1062591

[B19] Hattersley P. (1984). Characterization of C_4_ type leaf anatomy in grasses (Poaceae). Mesophyll: bundle sheath area ratios. *Ann. Bot.* 53 163–180. 10.1093/oxfordjournals.aob.a086678

[B20] Heimann L. Horst I. Perduns R. Dreesen B. Offermann S. Peterhansel C. (2013). A common histone modification code on C_4_ genes in maize and its conservation in sorghum and *Setaria italica*. *Plant Physiol.* 162 456–469. 10.1104/pp.113.216721 23564230 PMC3641223

[B21] Heintzman N. D. Hon G. C. Hawkins R. D. Kheradpour P. Stark A. Harp L. F. (2009). Histone modifications at human enhancers reflect global cell-type-specific gene expression. *Nature* 459 108–112. 10.1038/nature07829 19295514 PMC2910248

[B22] Hibberd J. M. Covshoff S. (2010). The regulation of gene expression required for C_4_ photosynthesis. *Annu. Rev. Plant Biol.* 61 181–207. 10.1146/annurev-arplant-042809-112238 20192753

[B23] Hibberd J. M. Quick W. P. (2002). Characteristics of C4 photosynthesis in stems and petioles of C3 flowering plants. *Nature* 415 451–454. 10.1038/415451a 11807559

[B24] Hou H. Wang P. Zhang H. Wen H. Gao F. Ma N. (2015). Histone acetylation is involved in GA-regulated sodCp gene expression in maize aleurone layers. *Plant Cell Physiol.* 56 2139–2149. 10.1093/pcp/pcv126 26374791

[B25] Hurkman W. J. Tanaka C. K. (1986). Solubilization of plant membrane proteins for analysis by two-dimensional gel electrophoresis. *Plant Physiol.* 81 802–806. 10.1104/pp.81.3.802 16664906 PMC1075430

[B26] Imaizumi N. Ku M. S. Ishihara K. Samejima M. Kaneko S. Matsuoka M. (1997). Characterization of the gene for pyruvate, orthophosphate dikinase from rice, a C_3_ plant, and a comparison of structure and expression between C_3_ and C_4_ genes for this protein. *Plant Mol. Biol.* 34 701–716. 10.1023/A:1005884515840 9278162

[B27] Ku M. Kano-Murakami Y. Matsuoka M. (1996). Evolution and expression of C_4_ photosynthesis genes. *Plant Physiol.* 111 949 10.1104/pp.111.4.949 PMC160960 8756491

[B28] Langdale J. A. (2011). C_4_ cycles: past, present, and future research on C_4_ photosynthesis. *Plant Cell* 23 3879–3892. 10.1105/tpc.111.092098 22128120 PMC3246324

[B29] Lea P. Chen Z.-H. Leegood R. Walker R. (2001). Does phosphoenolpyruvate carboxykinase have a role in both amino acid and carbohydrate metabolism? *Amino Acids* 20 225–241. 10.1007/s007260170041 11354601

[B30] Lee J.-S. Smith E. Shilatifard A. (2010). The language of histone crosstalk. *Cell* 142 682–685. 10.1016/j.cell.2010.08.011 20813257 PMC3711869

[B31] Leegood R. Ap Rees T. (1978). Phosphoenolpyruvate carboxykinase and gluconeogenesis in cotyledons of *Cucurbita pepo*. *Biochim. Biophys. Acta* 524 207–218. 10.1016/0005-2744(78)90119-5 656445

[B32] Leegood R. C. Walker R. (1999). “Phosphoenolpyruvate carboxykinase in plants: its role and regulation,” in *Plant Carbohydrate Biochemistry*, eds Bryant J. A. Burrell M. M. Kruger N. J. (Oxford: BIOS Scientific Publishers), 201–213. 10.1016/S0003-9861(03)00093-6

[B33] Li W. Lin Y.-C. Li Q. Shi R. Lin C.-Y. Chen H. (2014). A robust chromatin immunoprecipitation protocol for studying transcription factor–DNA interactions and histone modifications in wood-forming tissue. *Nat. Protocols* 9 2180–2193. 10.1038/nprot.2014.146 25144269

[B34] Ludwig M. (2012). Carbonic anhydrase and the molecular evolution of C_4_ photosynthesis. *Plant Cell Environ.* 35 22–37. 10.1111/j.1365-3040.2011.02364.x 21631531

[B35] Ludwig M. (2013). Evolution of the C_4_ photosynthetic pathway: events at the cellular and molecular levels. *Photosynth. Res.* 117 147–161. 10.1007/s11120-013-9853-y 23708978

[B36] Malone S. Chen Z.-H. Bahrami A. R. Walker R. P. Gray J. E. Leegood R. C. (2007). Phosphoenolpyruvate carboxykinase in Arabidopsis: changes in gene expression, protein and activity during vegetative and reproductive development. *Plant Cell Physiol.* 48 441–450. 10.1093/pcp/pcm014 17283014

[B37] Ni Z. Kim E.-D. Ha M. Lackey E. Liu J. Zhang Y. (2009). Altered circadian rhythms regulate growth vigour in hybrids and allopolyploids. *Nature* 457 327–331. 10.1038/nature07523 19029881 PMC2679702

[B38] Offermann S. Danker T. Dreymüller D. Kalamajka R. Töpsch S. Weyand K. (2006). Illumination is necessary and sufficient to induce histone acetylation independent of transcriptional activity at the C_4_-specific phosphoenolpyruvate carboxylase promoter in maize. *Plant Physiol.* 141 1078–1088. 10.1104/pp.106.080457 16679423 PMC1489893

[B39] Offermann S. Dreesen B. Horst I. Danker T. Jaskiewicz M. Peterhansel C. (2008). Developmental and environmental signals induce distinct histone acetylation profiles on distal and proximal promoter elements of the C_4_-Pepc gene in maize. *Genetics* 179 1891–1901. 10.1534/genetics.108.087411 18689888 PMC2516067

[B40] Okabe K. Yang S.-Y. Tsuzuki M. Miyachi S. (1984). Carbonic anhydrase: its content in spinach leaves and its taxonomic diversity studied with anti-spinach leaf carbonic anhydrase antibody. *Plant Sci. Lett.* 33 145–153. 10.1016/0304-4211(84)90004-X

[B41] Ong C.-T. Corces V. G. (2011). Enhancer function: new insights into the regulation of tissue-specific gene expression. *Nat. Rev. Genet.* 12 283–293. 10.1038/nrg2957 21358745 PMC3175006

[B42] Parsley K. Hibberd J. M. (2006). The Arabidopsis PPDK gene is transcribed from two promoters to produce differentially expressed transcripts responsible for cytosolic and plastidic proteins. *Plant Mol. Biol.* 62 339–349. 10.1007/s11103-006-9023-0 16915520

[B43] Peltier J.-B. Cai Y. Sun Q. Zabrouskov V. Giacomelli L. Rudella A. (2006). The oligomeric stromal proteome of *Arabidopsis thaliana* chloroplasts. *Mol. Cell. Proteomics* 5 114–133. 10.1074/mcp.M500180-MCP200 16207701

[B44] Perduns R. Horst-Niessen I. Peterhänsel C. (2015). Photosynthetic genes and genes associated with the C_4_ trait in maize are characterized by a new class of highly regulated histone acetylation peaks on upstream promoters. *Plant Physiol.* 168 1378–1388. 10.1104/pp.15.00934 26111542 PMC4528772

[B45] Podevin N. Krauss A. Henry I. Swennen R. Remy S. (2012). Selection and validation of reference genes for quantitative RT-PCR expression studies of the non-model crop Musa. *Mol. Breed.* 30 1237–1252. 10.1007/s11032-012-9711-1 23024595 PMC3460175

[B46] Rosche E. Westhoff P. (1995). Genomic structure and expression of the pyruvate, orthophosphate dikinase gene of the dicotyledonous C_4_ plant *Flaveria trinervia* (Asteraceae). *Plant Mol. Biol.* 29 663–678. 10.1007/BF00041157 8541493

[B47] Rylott E. L. Gilday A. D. Graham I. A. (2003). The gluconeogenic enzyme phosphoenolpyruvate carboxykinase in Arabidopsis is essential for seedling establishment. *Plant Physiol.* 131 1834–1842. 10.1104/pp.102.019174 12692343 PMC166940

[B48] Sage R. F. Sage T. L. Kocacinar F. (2012). Photorespiration and the evolution of C_4_ photosynthesis. *Annu. Rev. Plant Biol.* 63 19–47. 10.1146/annurev-arplant-042811-105511 22404472

[B49] Sheen J. (1999). C_4_ gene expression. *Annu. Rev. Plant Biol.* 50 187–217. 10.1146/annurev.arplant.50.1.187 15012208

[B50] Shi J. Yi K. Liu Y. Xie L. Zhou Z. Chen Y. (2015). Phosphoenolpyruvate carboxylase in Arabidopsis leaves plays a crucial role in carbon and nitrogen metabolism. *Plant Physiol.* 167 671–681. 10.1104/pp.114.254474 25588735 PMC4348777

[B51] Slack C. R. Hatch M. D. (1967). Comparative studies on the activity of carboxylases and other enzymes in relation to the new pathway of photosynthetic carbon dioxide fixation in tropical grasses. *Biochem. J.* 103 660–665. 10.1042/bj1030660 4292834 PMC1270465

[B52] Taniguchi M. Izawa K. Ku M. S. Lin J.-H. Saito H. Ishida Y. (2000). The promoter for the maize C_4_ pyruvate, orthophosphate dikinase gene directs cell-and tissue-specific transcription in transgenic maize plants. *Plant Cell Physiol.* 41 42–48. 10.1093/pcp/41.1.42 10750707

[B53] Taylor L. Nunes-Nesi A. Parsley K. Leiss A. Leach G. Coates S. (2010). Cytosolic pyruvate, orthophosphate dikinase functions in nitrogen remobilization during leaf senescence and limits individual seed growth and nitrogen content. *Plant J.* 62 641–652. 10.1111/j.1365-313X.2010.04179.x 20202167

[B54] Turner B. M. (2000). Histone acetylation and an epigenetic code. *Bioessays* 22 836–845. 10.1002/1521-1878(200009)22:9<836::AID-BIES9<3.0.CO;2-X 10944586

[B55] Valledor L. Pascual J. Meijón M. Escandón M. Cañal M. J. (2015). Conserved epigenetic mechanisms could play a key role in regulation of photosynthesis and development-related genes during needle development of *Pinus radiata*. *PLoS ONE* 10:e0126405 10.1371/journal.pone.0126405 PMC4429063 25965766

[B56] Walker R. P. Chen Z. H. Johnson K. E. Famiani F. Tecsi L. Leegood R. C. (2001). Using immunohistochemistry to study plant metabolism: the examples of its use in the localization of amino acids in plant tissues, and of phosphoenolpyruvate carboxykinase and its possible role in pH regulation. *J. Exp. Bot.* 52 565–576. 10.1093/jexbot/52.356.565 11373305

[B57] Walker R. P. Chen Z.-H. Técsi L. I. Famiani F. Lea P. J. Leegood R. C. (1999). Phosphoenolpyruvate carboxykinase plays a role in interactions of carbon and nitrogen metabolism during grape seed development. *Planta* 210 9–18. 10.1007/s004250050648 10592027

[B58] Walker R. P. Leegood R. C. (1996). Phosphorylation of phosphoenolpyruvate carboxykinase in plants. Studies in plants with C_4_ photosynthesis and Crassulacean acid metabolism and in germinating seeds. *Biochem. J.* 317 653–658. 10.1042/bj3170653 8760346 PMC1217536

[B59] Wang H. Alvarez S. Hicks L. M. (2011). Comprehensive comparison of iTRAQ and label-free LC-based quantitative proteomics approaches using two *Chlamydomonas reinhardtii* strains of interest for biofuels engineering. *J. Proteome Res.* 11 487–501. 10.1021/pr2008225 22059437

[B60] Wang P. Zhao L. Hou H. Zhang H. Huang Y. Wang Y. (2015). Epigenetic changes are associated with programmed cell death induced by heat stress in seedling leaves of *Zea mays*. *Plant Cell Physiol.* 56 965–976. 10.1093/pcp/pcv023 25670712

[B61] Westhoff P. Gowik U. (2004). Evolution of C_4_ phosphoenolpyruvate carboxylase. Genes and proteins: a case study with the genus *Flaveria*. *Ann. Bot.* 93 13–23. 10.1093/aob/mch003 14644912 PMC4242257

[B62] Wolffe A. P. Hayes J. J. (1999). Chromatin disruption and modification. *Nucleic Acids Res.* 27 711–720. 10.1093/nar/27.3.711 9889264 PMC148238

[B63] Xiao X. Ma J. Wang J. Wu X. Li P. Yao Y. (2015). Validation of suitable reference genes for gene expression analysis in the halophyte *Salicornia europaea* by real-time quantitative PCR. *Front. Plant Sci.* 5:788 10.3389/fpls.2014.00788 PMC4300904 25653658

[B64] Yu Q. Liu J. Wang Z. Nai J. Lü M. Zhou X. (2013). Characterization of the NADP-malic enzymes in the woody plant *Populus trichocarpa*. *Mol. Biol. Rep.* 40 1385–1396. 10.1007/s11033-012-2182-y 23096088

[B65] Zhou J. Wang X. He K. Charron J.-B. F. Elling A. A. Deng X. W. (2010). Genome-wide profiling of histone H3 lysine 9 acetylation and dimethylation in *Arabidopsis* reveals correlation between multiple histone marks and gene expression. *Plant Mol. Biol.* 72 585–595. 10.1007/s11103-009-9594-7 20054610

