# Supplementary material for: Histone Acetylation Modifications Affect Tissue-Dependent Expression of Poplar Homologs of C4 Photosynthetic Enzyme Genes
Source: Front Plant Sci. 2017 Jun 8;8:950. doi: 10.3389/fpls.2017.00950 (PMC5462996; doi:10.3389/fpls.2017.00950)
Supplement: Supplementary file 1 [file Data_Sheet_1.PDF]

Histone Acetylation Modifications Affect Tissue-Dependent Expression of Poplar Homologues of C_4 Photosynthetic Enzyme Genes

Yuan Li[1,2], Xiu-Mei Dong[1], Feng Jin[1], Zhuo Shen[1], Qing Chao[1], Bai-Chen Wang[1*]

Author Affiliation:

[1] Photosynthesis Research Center, Key Laboratory of Photobiology, Institute of Botany, Chinese Academy of Science, No.20 Nanxincun, Xiangshan, Beijing 100093, China

[2] State Key Laboratory of Forest Genetics and Tree Breeding, Northeast Forestry University, No.26 Hexing Road, Harbin 150040, China

liy080822@ibcas.ac.cn; dongxiumei@ibcas.ac.cn; jinfeng@ibcas.ac.cn; shenzhuo@ibcas.ac.cn; chaoqing@ibcas.ac.cn; wangbc@ibcas.ac.cn

Corresponding Author:

Bai-Chen Wang

Photosynthesis Research Center, Key Laboratory of Photobiology, Institute of Botany, Chinese Academy of Science, No.20 Nanxincun, Xiangshan, Beijing 100093, China

Tel: 8610-62836884

Fax: 8610-62836884

Email: wangbc@ibcas.ac.cn

SUPPLEMENTARY DATA

Supplemental Figure S1 Sequence alignment of poplar proteins homologous to C_4 photosynthetic enzymes in hybrid poplar. (A)-(D) Sequences were aligned with BioEdit software. (A) PsnCA proteins; (B) PsnPPDK proteins; (C) PsnPCK proteins and (D) PsnPEPC proteins.

Supplemental Figure S2 Selection of candidate reference genes for qRT-PCR studies of gene expression. (A) Cq values for the candidate reference genes. Cq values show the average level of gene expression in each tissue. Mean values are shown for at least three replicates per gene and tissue type. Bars indicate SE. (B) Average expression stability (M) values for the five candidate reference genes are shown. The stabilities of the candidate reference genes on the right are higher than those on the left.

Supplemental Figure S3 Chlorophyll content in different tissues of poplar. Chlorophyll a (chl a) and chlorophyll b (chl b) content in leaves (L), stem chlorenchyma (Sc), stem vascular tissue (Sv) and roots (R) of poplar. All tissues were harvested from five-month old poplar plants grown in a green-house. Mean chlorophyll content from six independent experiments is shown. Bars indicate SE.

Supplemental Figure S4 Location of the amplified segments (P1, P2, and P3) of the *PsnCA*, *PsnPPDK*, *PsnPCK* and *PsnPEPC* genes used in ChIP-qRT-PCR analysis.

Supplemental Figure S5 ChIP analysis of the *PsnACTIN2* control. (A) Location of the amplified segments (P1, P2, and P3) used in ChIP-qRT-PCR analysis. (B)-(D) ChIP analysis of three regions of *PsnACTIN2* promoter, including the distal region P1 (B), the middle region P2 (C) and the proximal region P3 (D). ChIP was performed with antibodies against H3K9ac antibody and H4K5ac antibody. The precipitates without using any antibody (NoAb) served as a negative control. The ChIP signal was quantified as relative to input DNA (% of IP). Leaves, Sc, Sv and roots of five-month old poplar plants grown in a green-house were collected for ChIP analysis. Values are the means from three independent experiments. Bars indicate SE.

Supplemental Figure S6 Selection of the candidate references genes in different tissues of poplar with TSA for two days. (A) Cq values for the candidate reference genes. Cq values show the average level of gene expression in each tissue of poplar

without TSA (control) and with TSA treatment. Mean values are shown for at least three replicates per gene and tissue type. Bars indicate SE. (B) Average expression stability of the candidate reference genes. Average expression stability (M) values for the five candidate reference genes results show the stabilities of the candidate reference genes on the right are higher than those on the left.

Supplemental Figure S7 Effect of TSA on H3K9ac and H4K5ac modification and expression of the *PsnCA*, *PsnPPDK*, *PsnPCK* and *PsnPEPC* genes in different tissues of poplar. (A) Western blot analysis of H3K9ac and H4K5ac protein levels in Sv of poplar treated with TSA for two days. ACTIN was used as a control for equal loading. (B-J) Expression of the *PsnCA*, *PsnPPDK*, *PsnPCK* and *PsnPEPC* genes in leaves, Sc, Sv and roots of poplar treated with TSA for two days. All transcription data were normalized to *PsnACTIN2* expression. Values are means from three independent experiments. Bars indicate SE.

Supplemental Figure S8 TSA affects the level of H3K9ac in the promoters of the *PsnCA*, *PsnPPDK*, *PsnPCK* and *PsnPEPC* genes in poplar. (A-I) ChIP was used to detect H3K9ac levels in the P3 (close to TIS) promoter of the *PsnCA*, *PsnPPDK*, *PsnPCK* and *PsnPEPC* genes in leaves, Sc, Sv and roots of poplar treated with TSA for two days. Values are the means from three independent experiments. Bars indicate SE. Asterisks indicated significantly different means (*$p<0.05$; **$p<0.005$) as determined with a t-test.

Supplemental Figure S9 TSA affects the level of H4K5ac in the promoters of the *PsnCA*, *PsnPPDK*, *PsnPCK* and *PsnPEPC* genes in poplar. (A-I) ChIP was used to detect H3K9ac levels in the P3 (close to TIS) promoter of the *PsnCA*, *PsnPPDK*, *PsnPCK* and *PsnPEPC* genes in leaves, Sc, Sv and roots of poplar treated with TSA for two days. Values are the means from three independent experiments. Bars indicate SE. Asterisks indicated significantly different means (*$p<0.05$; **$p<0.005$) as determined with a t-test.

Supplemental Table 1 Poplar genes homologous to C_4 photosynthetic enzyme genes

Supplemental Table 2 Primers used to amplify poplar genes homologous to C_4 photosynthetic enzyme genes

Supplemental Table 3 Primers used to amplify the promoters of poplar genes homologous to C_4 photosynthetic enzyme genes

Supplemental Table 4 Primers used in the selection of candidate reference genes

Supplemental Table 5 Primers used in qRT-PCR

Supplemental Table 6 Primers used in ChIP-qRT-PCR

Supplemental Figure S1

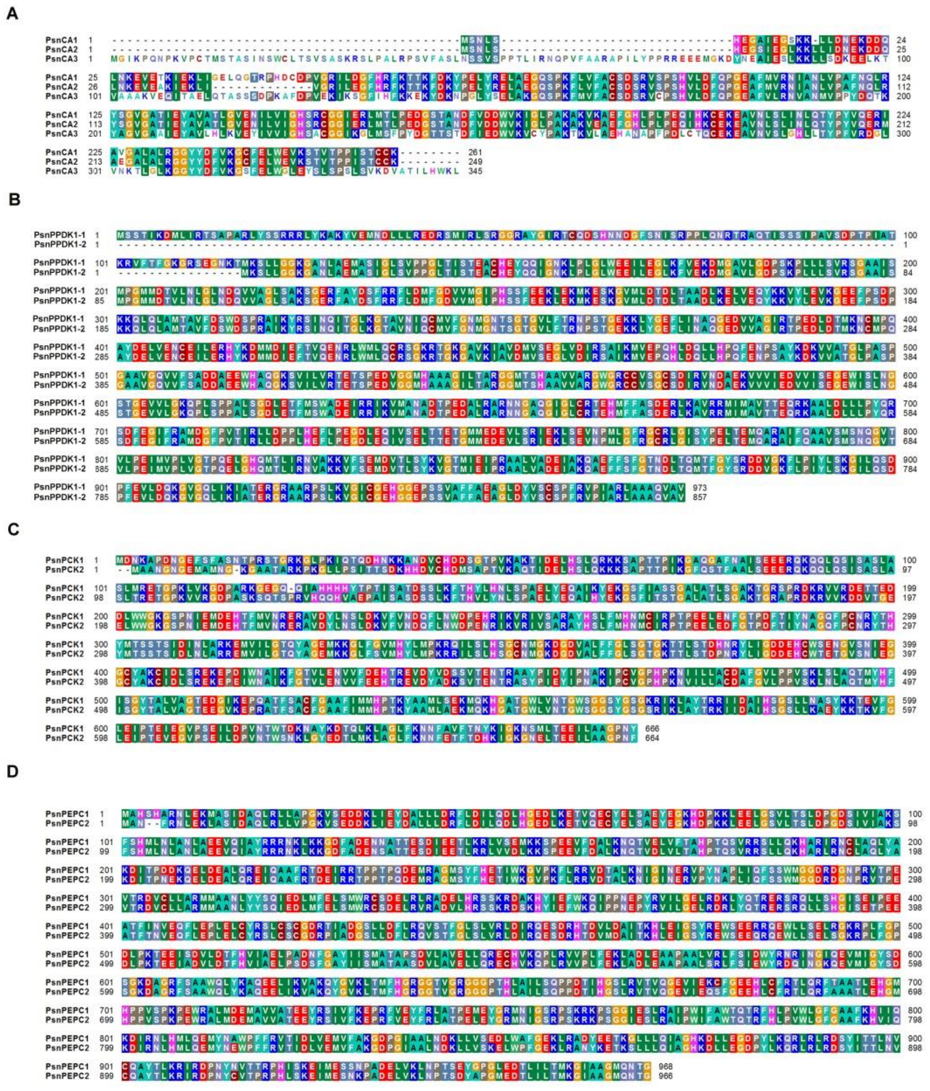

Supplemental Figure S2

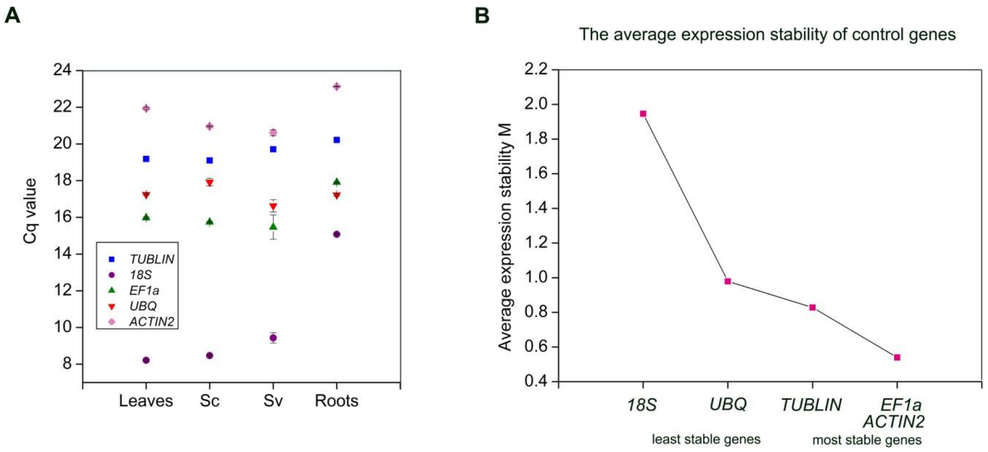

Supplemental Figure S3

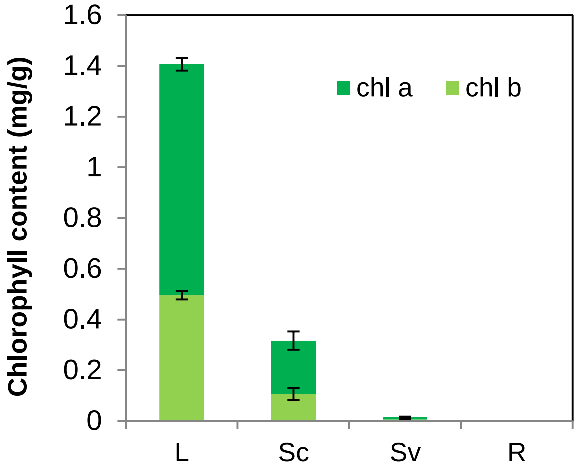

Supplemental Figure S4

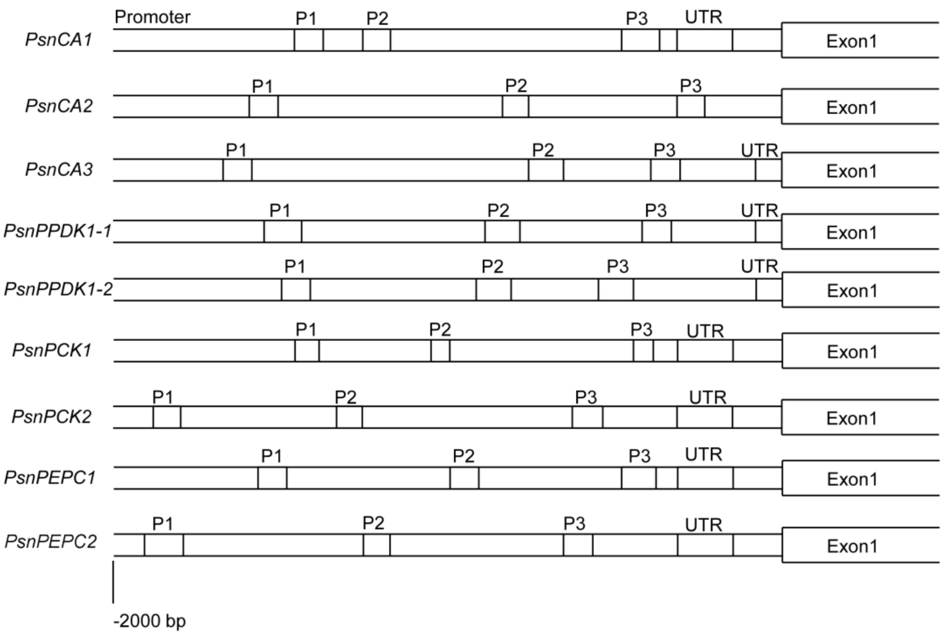

Supplemental Figure S5

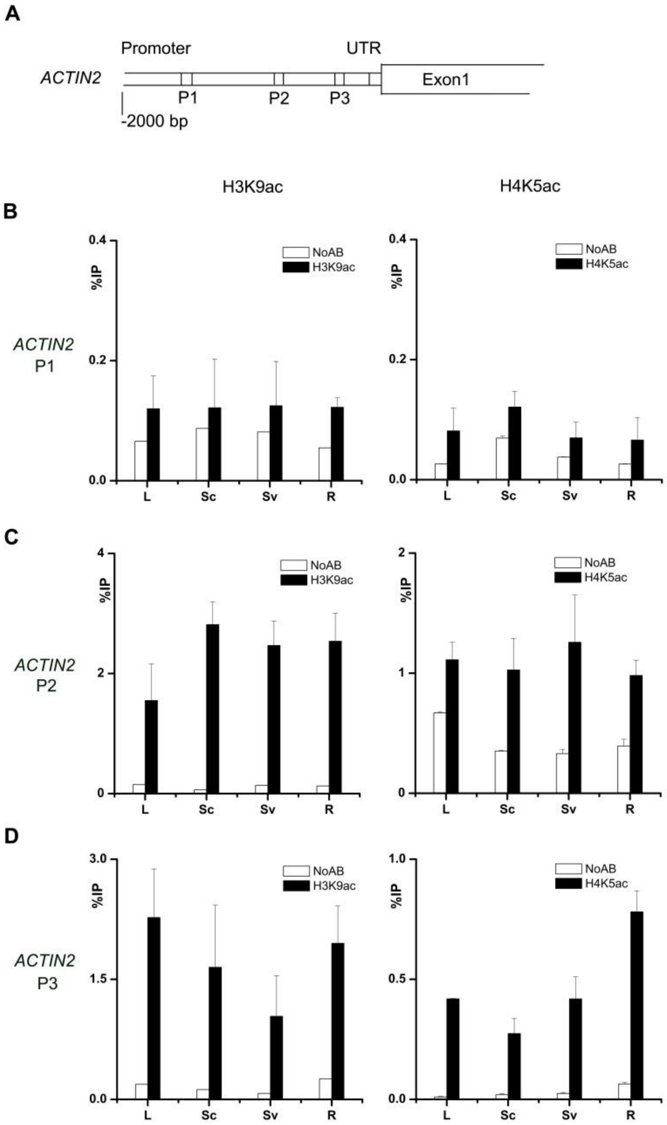

Supplemental Figure S6

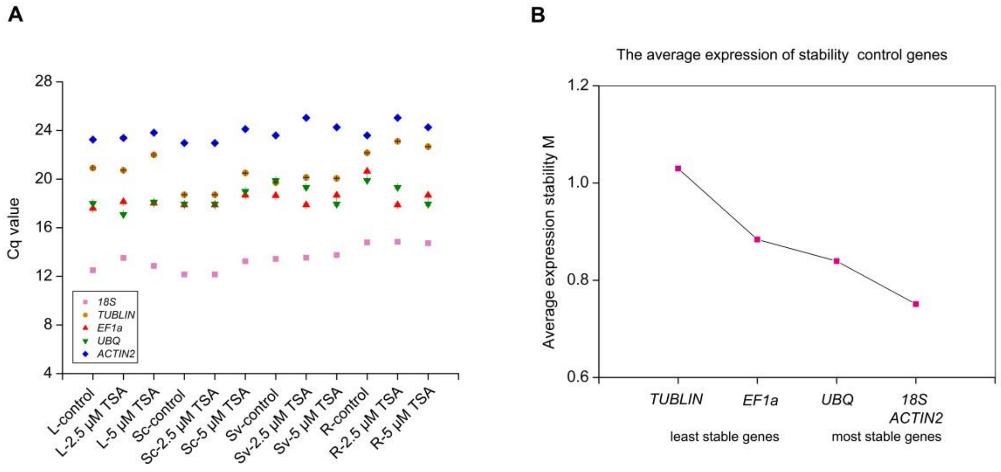

Supplemental Figure S7

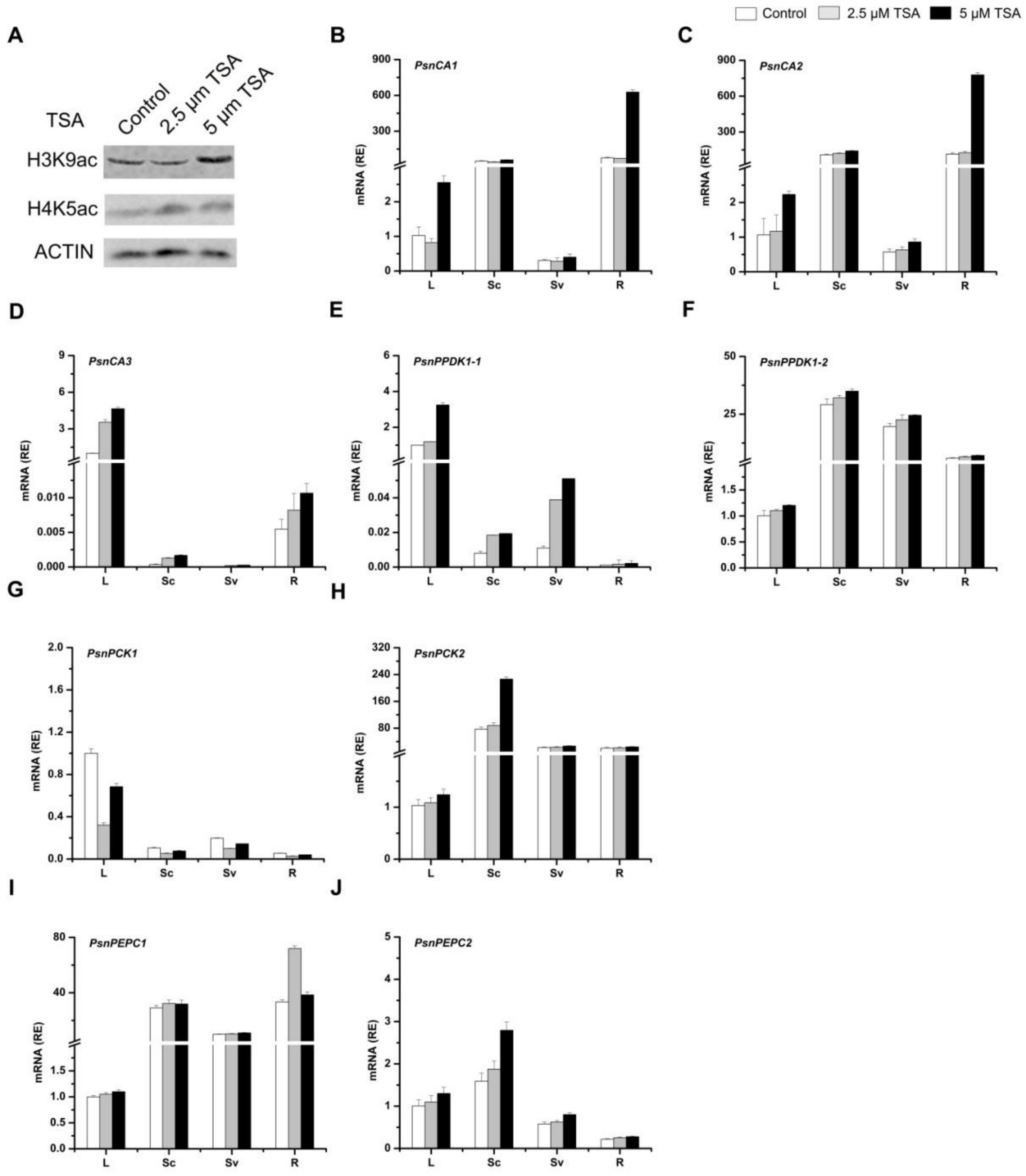

Supplemental Figure S8

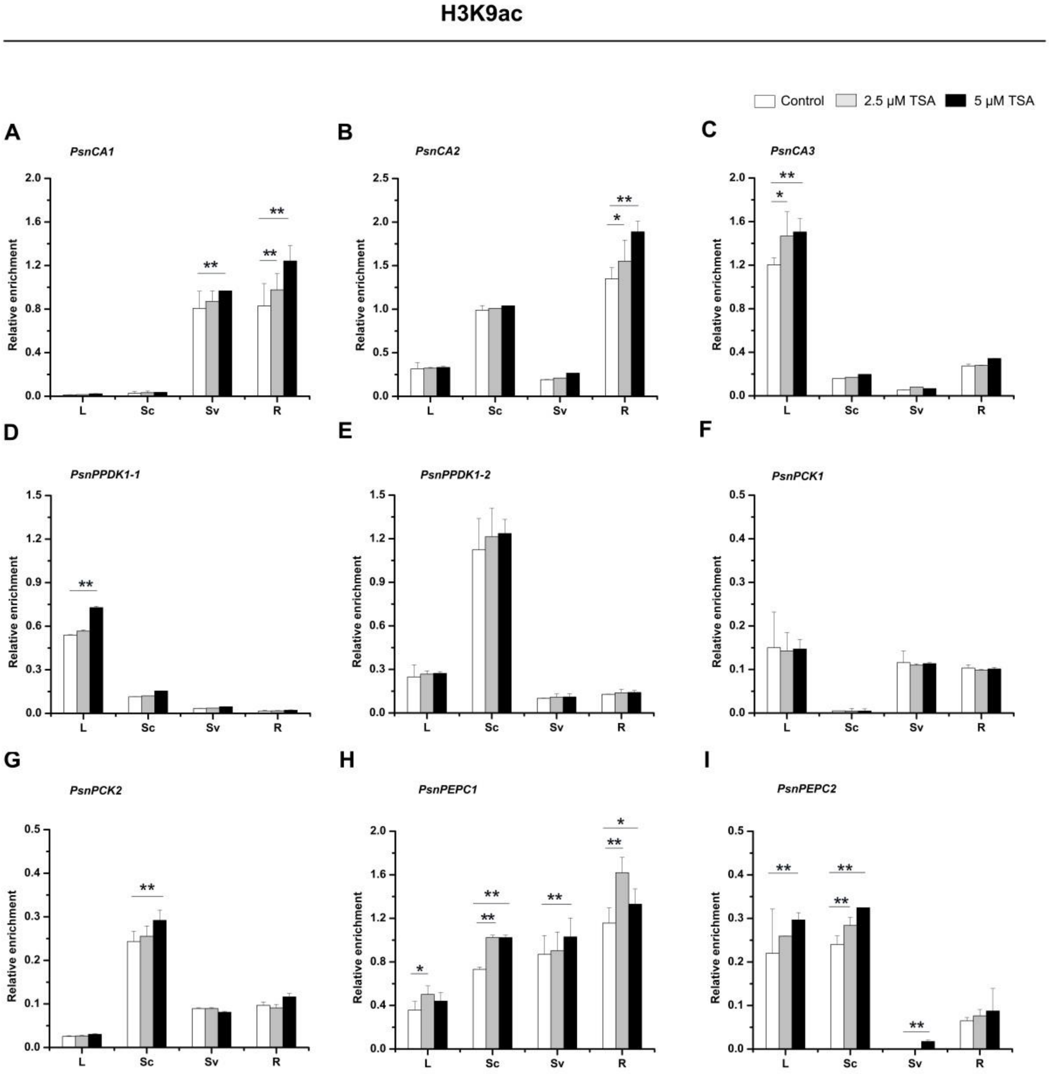

Supplemental Figure S9

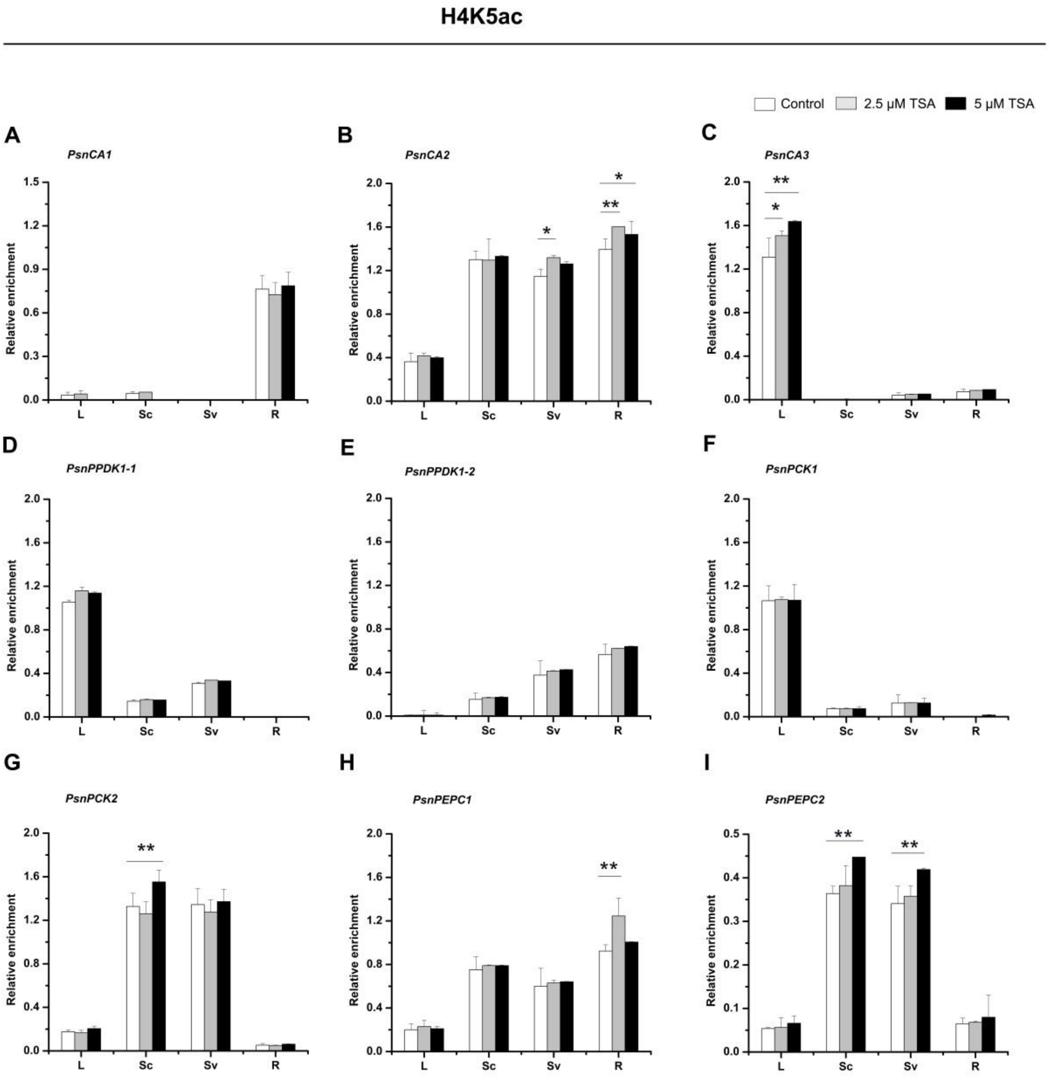

Supplemental Table 1

Gene name	Locus	Length (aa)
PsnCA1	Potri.015G076000.1	261
PsnCA2	Potri.015G075900.1	249
PsnCA3	Potri.001G348900.1	345
PsnPPDK1-1	Potri.010G027800.2	952
PsnPPDK1-2	Potri.010G027800.6	857
PsnPCK1	Potri.002G107700.4	666
PsnPCK2	Potri.007G011200.1	664
PsnPEPC1	Potri.001G391900.1	968
PsnPEPC2	Potri.011G110700.1	966

Supplemental Table 2

Primer Name	Sequence (5' → 3')
PsnCA1	CGGGATCCATGAGTAATCTGTCACATGAAGGGGC
	ACGCGTCGACCTTTGCAACACGTGGAGATGGG
PsnCA2	ATGAGTAATCTGTCACATGAAGGGT
	CTTGACCTATTTGCAACACGT
PsnCA3	GGAATTCCATATGATGGGTATAAAGCCACAGAATCC
	GGAATTCCAGCTTCCAATGTAGTATGGTG
PsnPPDK1-1	CGAATTCATGTCGTCAACGATAAAAGATATG
	GCTCGAGGAACATTAAAAAAAATCCTGTGTC
PsnPPDK1-2	ACTCATATCTTCTCTGATTTACGC
	GCGTAAATCAGAGAAGATATGAGT
PsnPCK1	GGAATTCCATATGATGGACAACAAGGCACCTGAC
	GGAATTCGTAATTAGGACCAGCTGCCAG
PsnPCK2	GGAATTCCATATGATGGCAGCAAACGGGAACGGAGAG
	CCCAAGCTTGAAATTTGGACCGGCTGCGAGG
PsnPEPC1	CGGAATTCATGGCTCACAGTCATGCTAGGAAT
	CCGCTCGAGCACCAGTGTTCTGCATGCCAG
PsnPEPC2	GGAATTCCATATGATGGCAAATTTTAGGAATTTAGAGAAG
	GGAATTCACCAGTGTTCTGCATGCCAG

Supplemental Table 3

Primer Name	Sequence (5' → 3')
18S rRNA	TCAACTTTCGATGGTAGGATAGTG
	CCGTGTCAGGATTGGGTAATTT
EF1a	GGCAAGGAGAAGGTACACAT
	CAATCACACGCTTGTCAATA
UBQ	GTTGATTTTTGCTGGGAAGCG
	GATCTTGGCCTTCACGTTGT
TUBLIN	CTTGATCGAGTGAGGAAGTTGGCTG
	ACGACTGCCGTTGAAACCTGAGGAG
ACTIN2	GCAGTCTTCCCCAGTATTGTT
	TCCCCAACATAGGCATCTTTC

Supplemental Table 4

Primer Name	Sequence (5' → 3')
PsnCA1	ATCTGTCACATGAAGGGGCT
	TTGAAGCGATGAAACCCCTC
PsnCA2	GCAACATCGCTAACCTGGTT
	ACCACAGCGACTATGTCCAA
PsnCA3	TAAGTACGCTGGAGTTGGGG
	AGGCCCTTAATTCCACCACA
PsnPPDK1-1	CAAGGCGAAGTATGTGGAAATG
	AGGTGGCCTGGAAATGTTAC
PsnPPDK1-2	AGGAAACAAGCTGCCACTAG
	GCCAGGCATTGAAATAGCAG
PsnPCK1	GGCACCTGACAATGGAGAGT
	ATCGTTTTGGCTTTCACTGG
PsnPCK2	GATGAATGGGAAAGGAGCTG
	CGCTCATATCATGGCAGACA
PsnPEPC1	TTACGGGCTGTTATCCTTTCC
	AGCCCCAAAGTTTAGACCAC
PsnPEPC2	TGGCATCACATATCAGCGTC
	AGATGCACGTAGAAACAGAGG
PsnACTIN2	GCAGTCTTCCCCAGTATTGTT
	TCCCCAACATAGGCATCTTTC

Supplemental Table 5

Primer Name	Sequence (5' → 3')
PsnCA1	CGCAAGCATTTTCAATTTTGT
	CATGTTAATTTCCTCGGGTGA
PsnCA2	CTAGTGTGGCACGCAAGCAT
	GCCTGTATATCTACATGAAATCAAAA
PsnCA3	TTTGGGCAGTATGATGTGGA
	GATGCTGCCTCTCAATCCTC
PsnPPDK1-1	AACTTGGGCTCAGTTCATGG
	TTGCCAGTCGATGATTACTTT
PsnPPDK1-2	GAGAAATTATTTGGATCGAATCAC
	TCGCTGCGTAAATCAGAGAA
PsnPCK1	CTGGTGGCTTTCATCGATTT
	CCAAATGCACTGGCTAATCC
PsnPCK2	TTGGCACTCTCACACAATCA
	TGTTCATGGCCACCTTGTTA
PsnPEPC1	TTCTTGATGCAAGAGGAATTTT
	GGATGAGATCGAGGGACAGA
PsnPEPC2	CCATAATTCCCTCAATTTCCA
	GTTTCAATCAAGCACGCAAA

Supplemental Table 6

Primer Name	Sequence (5' → 3')
PsnCA1-P1	AGACAAACCGATCTCTTCAAGG
	ATGTTTGAGAATGGTTGATGCTG
PsnCA1-P2	CATCTCGACTAATTCCACGGG
	CGAGTCTCACAAACCTCAGAG
PsnCA1-P3	GGATTACATGGTCGTTAACTTCAAG
	CACTAGGAATTATAAGCATGTGAACG
PsnCA2-P1	GCACGCAAGCATTTTCAAT
	TCAAACATCAATAATCCTCCACA
PsnCA2-P2	GGCCTTTGAAAACTAATTCCAGT
	GCCTTCAATCGTCAGTTTAGC
PsnCA2-P3	TTGTCAAGTATTCTCTCGTCCG
	CAACTTGTGCAGCAAATTCAAG
PsnCA3-P1	TGGTTGTGATGGTGATTGGA
	TGTGTAGCTAAATTTCTAAAAGCCC
PsnCA3-P2	TCAGCAATGGGAACGAATTTG
	ACAGAAAATGGCCTTACCTAGC
PsnCA3-P3	TGGTTCATCTTCATCCTCAGC
	CCCTTCAATAAATGCCAGACG
PsnPPDK1-1-P1	ACATAGATCTCCAAGTCCAGTTG
	ACCACGGTTCTAGCAAGTTATC
PsnPPDK1-1-P2	CTTTAAAATGTTCCCAAGGCCG
	CATCTCCCCATTATTTCTCCTCC
PsnPPDK1-1-P3	AAGTAGGGCAGATTCAAGCAG
	GAGTGTGATGGGAAATGAATTGG
PsnPPDK1-2-P1	AAGTAGGGCAGATTCAAGCAG
	GAGTGTGATGGGAAATGAATTGG
PsnPPDK1-2-P2	TTAGCTCCGCAATTCCTCAG
	GGCTAGAAGGAGTGTTAGGAAAG
PsnPPDK1-2-P3	ACCCGAGCACAAACTATCAG
	AACCATAACCAAACTCCCATCA
PsnPCK1-P1	AGGTGGGCTGAAACTCATTG
	TCGAAGACTCAAAGAGCCAC
PsnPCK1-P2	TGATCATTTCCCATCAGCCG
	TTTTGATCTGCACATGCGATG
PsnPCK1-P3	TTACAAGGAAGACATGGGTGAC
	GTCGAATTAAAACCAAAACAGTATGC
PsnPCK2-P1	AAATCTGGGTCCACCTCCAT
	TGTGAACGAAAATAATCTGTGGA
PsnPCK2-P2	CCACTTTCCAAACTTTCCTGTG
	GAAAGTGTTTTCCGTTGACCG

PsnPCK2-P3	TCCATTACACTACCAAACATCGG
	ATGAATAATATTAAGGCCCCGTTTG
PsnPEPC1-P1	AGCTTACCACCCGCTTTCTT
	CATTTGGTCCCTGTTGTTTG
PsnPEPC1-P2	CCTTTTCCGTTTCCTCTTGA
	ATGCTTTCGGAAATGCTGTT
PsnPEPC1-P3	TGCAACTTAACAACGAACATCAC
	TGTAGTGGAACTGTATATCATTACTCG
PsnPEPC2-P1	CCATAATTCCCTCAATTTCCA
	AGAAAAAGCATAGAGAGTTGGTCA
PsnPEPC2-P2	AGGTGGAAGAGTTGTTGGTG
	CTCCTGAGATTGGTGATCGTC
PsnPEPC2-P3	GCGGCATATCACGGTATAGAAG
	CTCTCGGCTTTCACTCTTTACT
PsnACTIN2-P1	GCCAGAACAACTGGAAGTAAGA
	GGGAAGTGAAGAAGGCTTTGA
PsnACTIN2-P2	GAAGGATCCCAGCGTGTATATAAT
	GGCTGGTTCATTTGGCAATAAG
PsnACTIN2-P3	CAAAGTAAAGCAAGGCGTCAAG
	AGAGAGAAGAGAGGGAACAGG
